# Microbial diversity mediates the impact of air pollution on pneumococcal disease risk

**DOI:** 10.64898/2025.12.10.25341877

**Published:** 2025-12-11

**Authors:** Sophie Belman, Cebile Lekhuleni, Jackie Kleynhans, Giovenale Moirano, Daniela Lührsen, Cristina Carnerero, Susan Meiring, Stephanie W. Lo, Mignon du Plessis, Anne von Gottberg, Rachel Lowe

**Affiliations:** 1) Barcelona Supercomputing Center (BSC), Barcelona, Spain; 2) Parasites and Microbes, Wellcome Sanger Institute, Hinxton, UK; 3) Centre for Respiratory Diseases and Meningitis, National Institute for Communicable Diseases of the National Health Laboratory Service; Johannesburg, South Africa; 4) Department of Clinical Microbiology and Infectious Diseases, School of Pathology, Faculty of Health Sciences, University of the Witwatersrand, Johannesburg, South Africa; 5) School of Public Health, Faculty of Health Sciences, University of the Witwatersrand, Johannesburg, South Africa; 6) Division of Medical Microbiology, Department of Pathology, Faculty of Health Sciences, University of Cape Town, Cape Town, South Africa; 7) Catalan Institution for Research and Advanced Studies (ICREA), Barcelona, Spain; 8) London School of Hygiene and Tropical Medicine, London, UK; 9) Department of Medical Sciences, University of Turin, Italy; 10) Division of Public Health Surveillance and Response, National Institute for Communicable Diseases of the National Health Laboratory Service; Johannesburg, South Africa

## Abstract

*Streptococcus pneumoniae* (pneumococcus) remains a major global cause of invasive human disease with clear seasonal variation suggesting a role for environmental factors. The pneumococcus includes >100 serotypes and many lineages with different disease patterns. Interactions between microbial diversity, environmental factors, and invasive pneumococcal disease (IPD) remain unclear. We examined the effects of air pollution and meteorological factors on 59,017 IPD cases over 15 years, including 4,350 genome-sequenced isolates, using Bayesian spatio-temporal hierarchical models that link environmental exposures to bacterial diversity. Humidity and air pollution conferred the highest disease risk. In South Africa low humidity increased risk while high humidity was protective. Weekly PM_2.5_ 50 μg/m^3^ was associated with 4% cumulative risk increase with diversity-specific lag structures suggesting partially vaccine-mitigated risk. PM_2.5_ risk was greatest in adults ≥65 and varied by disease type. Understanding these relationships may refine vaccine-impact models and inform air pollution policy targets.

## Introduction

Streptococcus pneumoniae (the pneumococcus) is asymptomatically carried in the human nasopharynx and can cause both non-invasive diseases (such as otitis media and sinusitis), and invasive pneumococcal disease (IPD) such as bacteraemia and meningitis, when the bacterium breaches the epithelium or enters the bloodstream^1,2^. The progression from colonization, to asymptomatic carriage, to invasive disease is mediated by a combination of environment, microbial virulence, and host immunity. The pneumococcus has hundreds of known genomic lineages with hundreds of years of diversity among them. Here we use Global Pneumococcal Sequence Clusters (GPSCs) to classify pneumococcal lineages. The pneumococcus is typically enclosed in a polysaccharide capsule of which there are more than 100 known serotypes. The seven-valent and 13-valent pneumococcal conjugate vaccines (PCV7 and PCV13) target seven and 13 serotypes respectively. This has resulted in serotype replacement by non-vaccine serotypes (NVTs) in the years following vaccine implementation globally^3–6^.

Despite high vaccination coverage, IPD shows consistent seasonal spikes each year in part due to the extensive diversity of the pneumococcus. Efforts to reduce residual IPD burden is focused on adapting vaccination strategies to settings with varied sociodemographic contexts and pneumococcal colonization densities^7–10^. However, the role of air quality and meteorological factors, such as air pollution, temperature and humidity in pneumococcal colonization and disease dynamics is typically overlooked. Many geographic regions with the highest pneumococcal disease burden also have populations with extensive exposure to polluted air^14^. Understanding the sensitivity of pneumococcal dynamics to air quality and meteorological factors could allow vaccination strategy modeling and winter hospital capacity preparations to better adapt to different geographies and changing climates. Accounting for underlying pneumococcal diversity when studying the role of environmental factors in driving IPD transmission and incidence might be crucial given the known variability in virulence, carriage durations, and sociodemographic populations affected by different lineages and serotypes. By accounting for pneumococcal diversity we aim to additionally uncover the biological mechanisms behind environment-microbe interactions.

Here we focus on particulate matter <2.5μm in size (PM_2.5_), and the associated risk for IPD while accounting for the underlying microbial diversity as well as additional environmental factors. We investigated the role of PM_2.5_ in influencing IPD incidence using IPD case data spanning 2005 to 2023 (incorporating genomic data in models through 2019), from 531 hospitals in South Africa. This is a setting with pneumococcal colonization estimates ranging from around 40–60% (typically measured in under 5 year-olds)^15,16^, and marked geographic and climatic diversity. Major public health interventions occurred during the study period (PCV7 introduction in 2009, PCV13 in 2011, and widespread antiretroviral therapy (ART) for treatment of people living with HIV (PLHIV), since 2005). We include underlying pneumococcal phenotypic and genomic data to understand interactions with underlying microbial ecology, accounting for different serotypes and genomic lineages. We apply Bayesian hierarchical models to capture spatial and temporal variability, incorporate lagged relationships between environmental exposure and disease onset, and allow for stratification by age group and disease type. These models enable a better understanding of the interaction between pneumococcal populations and environmental factors which plays a role in IPD morbidity and mortality.

## Results

**Figure 1.**
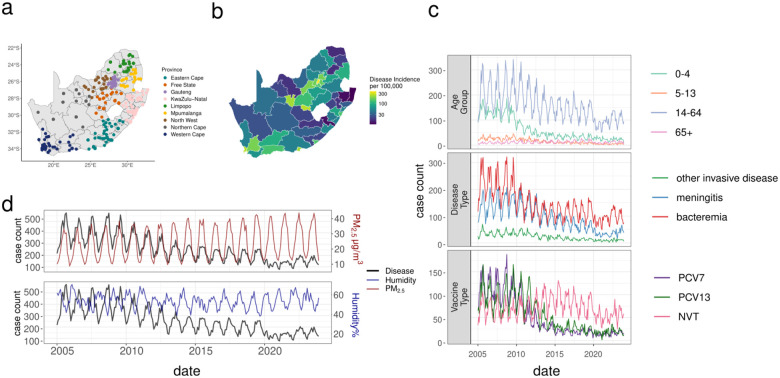
Summary of epidemiological and environmental datasets in South Africa. (a) Hospital locations (N=531) colored by province (N=9) (b) Log10 incidence rate per 100,000 from 2005–2023 by district (N=52) in South Africa (c) monthly case counts by age group, disease type (bacteremia, meningitis, other IPD types), vaccine type (PCV7 = seven-valent vaccine type serotypes; PCV13 = additional six serotypes in the 13-valent vaccine type; NVT = non-vaccine types), and serotype (colored by PCV7, PCV13, and NVT). (d) monthly pneumococcal disease case counts (black) with mean monthly particulate matter <2.5 μm concentration (red), and mean relative humidity (blue) across South Africa over 18 years; left axis is the monthly case counts, right axis is the concentration (μg/m^3^) of particulate matter <2.5μm (upper panel) and percent relative humidity (lower panel).

A total of 59,017 identified IPD cases were included from 531 hospitals in South Africa between 2005–2023. The majority of cases were found in urban centers where the majority of the South African population resides (Figure 1A–B). These included bacteremia, meningitis, and other IPD types. The majority of cases occurred in people aged 15–64 years old (62%), followed by children under 6 years of age (23%). 4.8% of cases were from over-64 year olds. There was a well characterized decrease in serotypes that were targeted by PCVs. We observed NVT persistence (Figure 1C) and consistent seasonality in IPD cases. Cases peaked in colder months across the time period coinciding with the seasonality of PM_2.5_ concentration and inversely correlating with relative humidity (Figure 1D)(Figure S1)(Table S1).

### Seasonal, interannual, and spatial effects

**Figure 2.**
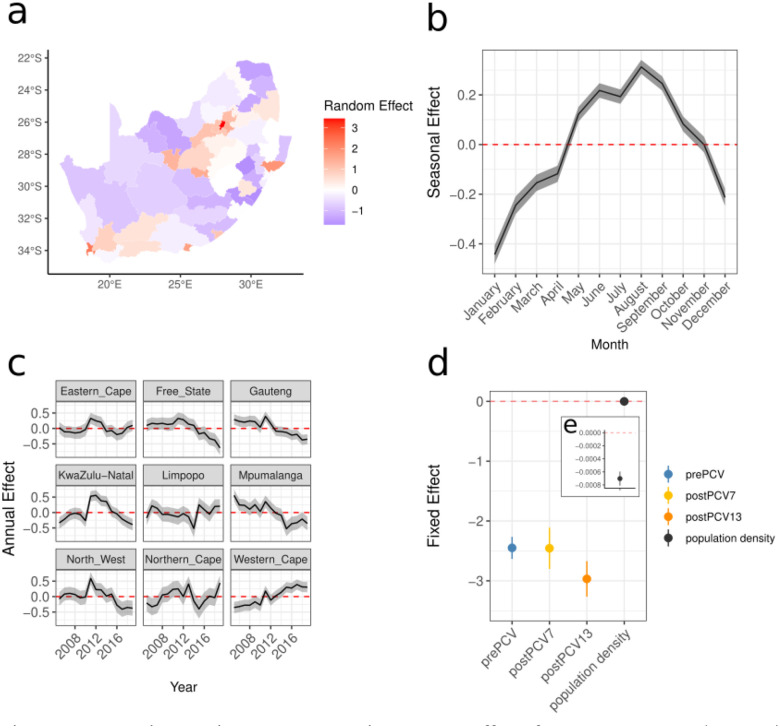
**Evaluating spatial, seasonal, and interannual effects for a base model** and comparing interventions (a-c) Random effects for the base model including spatial, seasonal, and interannual effects as well as accounting for implementation of the vaccine in 2009, 2011, and population density (a) spatially across 52 districts where a positive effect (more cases than expected on average) is red and a negative effect is blue, (b) seasonally across the 12 months of the year, the red line denotes effect size of 0, (c) across each of 2005–2019 included in the model replicated across the 9 provinces of South Africa (d) fixed effects for each vaccine period prePCV (2005–2008) (blue), postPCV7 (2009–2011) (yellow), and postPCV13 (2012–2019) (orange), and for the population density (black) (e) an inset of the population density effect.

Our baseline model (M12) included spatial autocorrelation across districts, seasonal, and interannual random effects replicated across provinces; while also accounting for the pneumococcal vaccine implementation and population density (Table 4). We found similar effects for models spanning 2005–2023 and 2005–2019 but due to the dramatic decrease in IPD cases with the pandemic period we proceeded with models restricted to the 2005–2019 period throughout (Table S2–3). The baseline models were fitted to weekly invasive disease incidence across South Africa both at district (N=52) and province levels (N=9). The Gauteng and Western Cape Provinces had a higher risk of IPD than the national average. The relative risk (RR) derived from the spatial effect for Gauteng Province was 13.35 (95% credible intervals (cis) 4.19–44.34) and for Western Cape Province was 1.54 (95% cis 0.84–2.70), representing the area specific risk relative to the average across all provinces. The risk in Western Cape Province was largely driven by the increased risk in the Cape Town district alone (relative to all district average)(RR: 10.16; 95% cis 7.89–13.09). All districts in Gauteng Province exhibited elevated disease risk relative to the national average (RR>1). Some of the exacerbated risk in the main cities may reflect travel to hospitals from more rural areas (Figure 2A)(Figure S1C). Across the other provinces, districts which host major cities and transport hubs had twice the risk of IPD compared to average (Figure 2A)(Table S3). There was a seasonal effect with an increased disease risk between the months of May and October with peak risk in August (RR: 1.37; 95% cis 1.33–1.41). In the interannual effect we found a reduction in risk over time even while accounting for vaccination (Figure 2C). This may be attributed to uptake of ART (Figure S1D).

There was a correlated increase in coverage for ART and vaccination across the study period (Pearson *R*^*2*^ = 0.84) and their inclusion in the models reduced interannual effect variance (Figure S1D)(Figure S2A). We found no effect from ART or PCV on NVT serotypes alone. Interestingly, models estimating NVT disease (in this high HIV burden setting)^17^ had substantial impacts on IPD when we include years 2020–2023 (Figure S2C–D). This may reflect the increase in cases among adults ages 18–64 after the SARS-CoV-2 reductions in 2020 and waning vaccine effect since the rapid uptake (Figure S2E)^18–20^. Due to the established direct relationship between pneumococcal vaccination and IPD, and the nuanced impact of ART, we included the PCV periods as the intervention-covariate in the baseline model (Figure 2D)(Table S4).

### Contribution of environmental factors to pneumococcal disease risk

We fitted the same baseline model as above, iteratively, with univariable environmental factors to estimate the risk of IPD across an 8-week time period from environmental exposure (M12, Table 4). We used distributed lagged non-linear models (DLNMs) to incorporate environmental factors (Figure S3). Model selection based on WAIC from univariable models, indicated that relative humidity, precipitation, PM_2.5_ and particulate matter <10μm (PM_10_), sulfur dioxide (SO_2_), and maximum temperature had the best fit. We estimated the risk of disease above the 2016–2029 NAAQS thresholds^21^ for PM_2.5_ (40 μg/m^3^), PM_10_ (75 μg/m^3^), and SO_2_ (125ug/m^3^).

Humidity and precipitation were negatively associated with IPD risk, whereas PM_2.5_, PM_10_, and SO_2_ were positively associated with IPD risk (cumulative 8-week effect). Maximum temperatures below 20°C and above 30°C were negatively associated with IPD, and maximum temperatures from 25–30°C were associated with an acute increased risk (in the first 1–2 weeks). These patterns persisted when models were fitted to Gauteng alone (Figure S4)(Figure S5)(Table S5).

**Figure 3.**
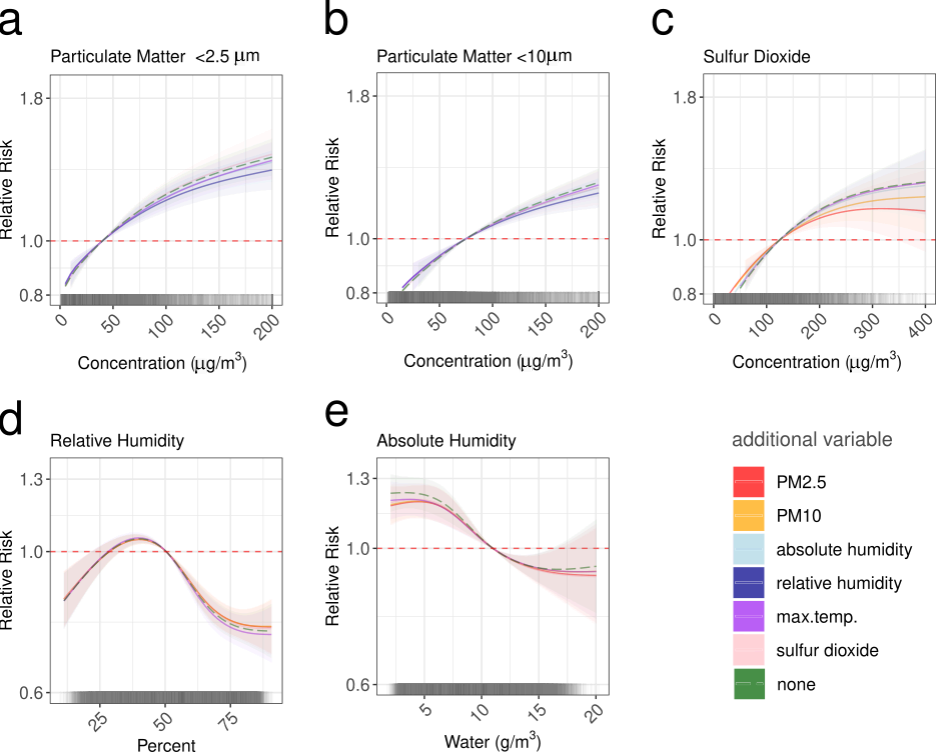
Cumulative relative risk across 8-weeks of lags for PM_2.5_, PM_10_ SO_2_, relative, and absolute humidity with additional multiplicative variables. In the DLNM the humidity variable cross basis has 3-degrees of freedom (df), while the pollution variables have 2. The additional multiplicative effects are included as fixed effects except the humidity variables which are non-linear with 5 cuts. Each plot includes the exposure-response curve for each lag-week colored by the multiplicative variable for (a) PM_2.5_ (models for none, absh, hurs, tasmax, SO_2_) (b) PM_10_ (models for none, absh, hurs, tasmax, so2), (c) SO_2_ (models for none, PM_2.5_, PM_10,_ absh, hurs, tasmax) (d) % relative humidity (models for none, PM_2.5_, PM_10_, SO_2_, tasmax), and (e) absolute humidity (models for none, PM_2.5_, PM_10_, SO_2,_ tasmax). This model is fit to weekly district level data from 2005–2019 across South Africa. At the bottom of each is a rug plot with the distribution of the main variables.

To understand the effect of PM_2.5_ and PM_10_, together with other environmental factors, we also explored the multiplicative effects of including multiple environmental variables in a single model. There was a higher risk of IPD between 35% and 47% relative humidity, which remained consistent after accounting for PM_2.5_ or PM_10_ as a multiplicative effect. Relative humidity of 39.8% had the highest IPD risk (RR = 1.05; 95% CI: 1.03–1.07)(Figure 3C). There was a strong protective effect of high relative humidity with a 25% decreased risk of pneumococcal disease at 74.8% relative humidity (RR: 0.77; cis 0.73–0.80) (Figure 3E). The risks from relative humidity waned after about four weeks. The acute disease risk with low absolute humidity (which in contrast with relative humidity does not account for the temperature-dependent variation in the air’s capacity to retain water) was strongly attenuated by PM_2.5_ and PM_10,_ however, with a 2-week lag time the risk from low absolute humidity was 1.23 (95% cis 1.16–1.31)(Figure S6)(Table S5).

With a weekly average of 50 μg/m^3^ of PM_2.5_ there was a 4% 8-week cumulative increased risk of IPD (RR 1.04 [95% cis 1.03–1.5]), but accounting for relative humidity decreased that risk to 3.6%. Strikingly, in this model there was a 21% increased IPD risk (1.21: 95% cis 1.16–1.27) with a weekly PM_2.5_ of 100 μg/m^3^ (Figure 3A)(Figure S6A)(Table S5). The effects of PM_2.5_ were immediate but the maximum risk was in the fourth week after exposure. The City of Johannesburg regularly exceeded weekly averages of 100 μg/m^3^ of PM_2.5_ (32 weeks in 2023) and across South Africa the weekly PM_2.5_ concentration was at least 50 μg/m^3^ in about 23 districts a year (about 14 weeks per district)(Figure S7)(Table S5). In 2019 Gauteng Province had a mean PM_2.5_ concentration of 117 μg/m^3^ and 718 observed IPD cases, and the population attributable fraction, based on weekly PM_2.5_ concentrations (accounting for relative humidity), and given causal assumptions in our modelled exposure–response relationship, was 2.06% (14.8 IPD cases) meaning if PM_2.5_ had been maintained at or below 40 μg/m^3^ 14.8 cases could theoretically have been averted; if this were reduced further, to the more stringent WHO threshold of 15 μg/m^3^, 24.3 cases (3.39%) would be averted. A weekly PM_10_ concentration of 100 μg/m^3^ increased the cumulative 8-week risk of disease 6.5% (RR 1.07: 95% cis 1.05–1.08) and similarly to SO_2_ the effect was lagged with an increased risk of disease only 3–8 weeks after exposure (Figure S6).

### Risk from PM_2.5_ varies by age, disease, and pneumococcal serotype

In the same environmental model formulations as above, we stratified outcome by particular demographic groups, disease outcomes, or pneumococcal serotypes. We ran each model with univariable DLNMs for PM_2.5_, PM_10_, SO_2_, and relative humidity. Model performance varied but PM_2.5_ and relative humidity had consistently good overall model performance (Table S5).

#### Age and disease outcome with PM_2.5_

**Figure 4.**
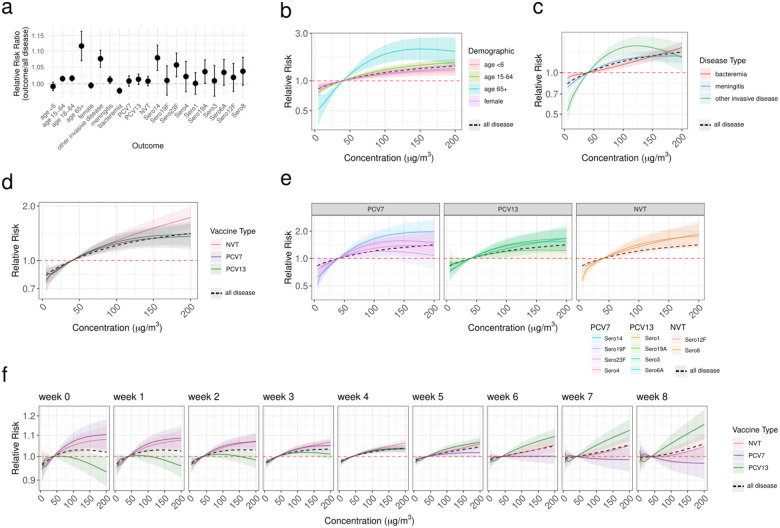
**Risk of specific sub-outcomes of invasive pneumococcal disease given weekly averages of particulate matter <2.5μm (PM_2.5_)** in a baseline model including population density and vaccination period together with random effects at the district level from 2005–2019. (a) reporting the relative risk ratio of each specific model compared to the model including all disease at 50 μg/m^3^ (exceeding the NAAQS threshold for PM_2.5_ by 10 μg/m^3^) (b) across age groups including age ≤5 (red), age 15–64 (green), and age 65 and older (blue), and including only females (purple), (c) the relative risk of PM_2.5_ across disease types for bacteremia (red), meningitis (blue), and other invasive disease types (green), (d) models for disease caused by serotypes targeted by PCV7 (purple), additional 6 serotypes in PCV13 (green), and NVT (red) (e) and fitting models to disease caused by the particular serotypes in these groups faceted by vaccine type and colored by serotype. (f) the relative risk with a 0 to 8 week lag time from PM_2.5_ to disease for each vaccine type group (pcv7=purple, pcv13=green, nvt=red). All models also include the RR from a model including all disease cases (black dashed).

The highest risk from PM_2.5_ was for those aged ≥65 years, with a cumulative RR across 8 weeks, and 50 μg/m^3^ weekly average PM_2.5_ exposure was 1.16 (cis 1.10–1.22)(Figure 4B). The relative risk ratio between each subset model and the main model (all IPD) translated to a relative risk ratio of 1.12 (95% cis 1.07–1.17) indicating a 12% higher risk for this age group at this PM_2.5_ concentration (Figure 4A). Those between the ages of 15–64 had a more acute impact with a RR>1 in the week of high PM_2.5_ concentrations (lag 0) while all other demographic groups showed similar lag patterns with the highest risk 3–4 weeks after exposure. This may reflect the higher prevalence of HIV among this age group^17^. The risk for those ≤5 years old was not negligible. For children ≤5 years, at 50 μg/m^3^ weekly average there was a 8-week cumulative RR of 1.03 (cis 1.01–1.05), and for females of all ages it was 1.04 (cis 1.02–1.05). While similar to the average risk of IPD across the population it translates to 3% more children with IPD at high pollution levels and highlights that PM_2.5_ does not only pose an increased disease risk for adults. (Figure S8). Models for particular disease outcomes (bacteremia, meningitis, or other invasive disease) had similar risk patterns. However, there was a higher cumulative risk (RR 1.12: 95% cis 1.08–1.16) and a more acute risk (>1 at early lag weeks) for other invasive disease types (Figure 4C)(Figure S8). The risk with a 50 μg/m^3^ weekly average was higher for meningitis (RR 1.05: 95% cis 1.03–1.07) than for bacteremia (RR 1.02: 95% cis 1.00–1.04)(Figure 4C), possibly indicating different mechanisms of effect across IPD types.

#### Serotype and vaccine type with PM_2.5_

The cumulative risk from PM_2.5_ across all vaccine types was similar across the 8 weeks, there was a varied lag pattern with an immediate effect for NVTs and PCV7 serotypes but a longer time to effect for the additional serotypes in PCV13 (Figure 4D–E). These effects persisted when we stratified by pre- and post-PCV periods implying a difference in mechanism not just changing serotype proportions (Figure S9). When further stratified by specific serotypes variable underlying dynamics emerged. With an exposure of 50 μg/m^3^ of PM_2.5_ weekly we found a relative risk ratio of >1 as compared to on average in the population for PCV7 serotypes 14 and 23F, and it trended higher for NVT serotype 8 and PCV13 serotype 19A (Figure 4A)(Figure 4E)(Figure S10A–B). We found acute risks from PM_2.5_ for NVT serotype 8 (94% GPSC3 in this dataset), and PCV13 serotypes 23F (68% GPSC14) and serotype 4 (58% GPSC70). These are all known invasive types. The 8-week cumulative risk for serotype 14 disease with 50 μg/m^3^ average weekly PM_2.5_ exposure was greater than on average in the population (RR ratio: 1.08 95% cis 1.04–1.12)(Figure 4A). Serotype 14 is targeted by PCV13 and in South Africa predominantly is represented across several different lineages including the lineages GPSC10 (49%), GPSC18 (25%), and GPSC9 (20%)(Figure S10C). The predominantly lagged effect of PM_2.5_ on vaccine serotypes demonstrates a protective effect from the vaccine against some of the immediate risks conferred from high levels of PM_2.5_ for IPD.

### Timing of air quality effects is mediated by lineage diversity

We estimated the impact of particular GPSC-lineage prevalence on the PM2.5 effect on IPD at the province level, accounting for a 8-week lag time from the years 2005–2019. In these models we include IPD as the outcome but to incorporate genomic lineages into the model (N=4350) we included the proportion sequenced at each space-time increment as an effect modifier (Figure S11). We interacted the crossbasis for each environmental factor with the proportion of each dominant genomic lineage (N>=90 as well as GPSC8) among sequenced isolates (Figure S12)(Table S1). Models with GPSC41 and GPSC8 had worse goodness-of-fit metrics than the model which excluded lineage diversity interaction effects (Figure S13)(Table S5).

**Figure 5.**
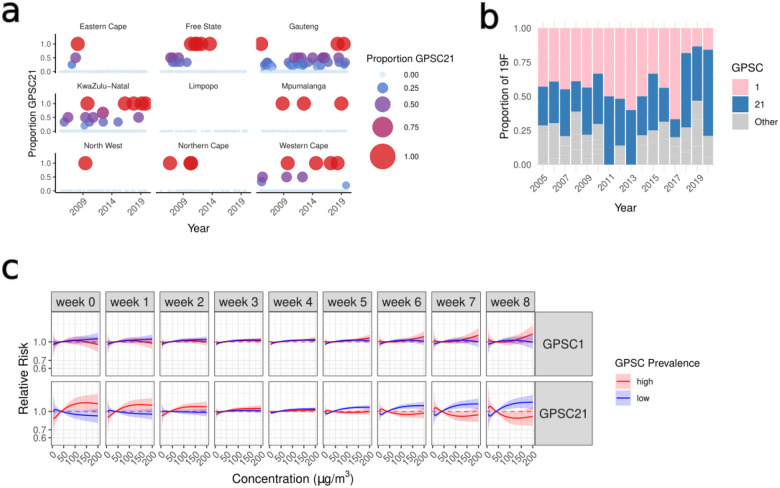
**Incorporating GPSC21 by modifying the effect of PM_2.5_ on IPD** by the (a) proportion of sequenced which were GPSC21 in each week and province (b) The proportion of serotype 19F isolates which are found on each GPSC per year highlighting GPSC1 (pink) and GPSC21 (blue) (c) exposure-response curves for the relative risk across concentrations of PM2.5 for GPSC1 (top) and GPSC21 (bottom) including the curves for high prevalence (red) and low prevalence (blue) of each lineages.

We explored the risk of IPD given the GPSC prevalence across concentrations of PM2.5 (where prevalence is across quantiles of GPSC proportions over the entire time period; low=0.1 and high=0.9)(Figure 5A)(Table S6). While the 8-week cumulative effect did not vary with lineage diversity there was variation in the risk across lags and GPSCs (Figure S13A–B). There was an acute effect for GPSC21 whereby the risk of IPD was elevated in time-space increment with high prevalence of these lineages. The cumulative effect with a GPSC21 interaction also had different exposure-response curves between high and low prevalence (Figure 5C). We found an early effect whereby locations with a high prevalence of GPSC21 had an acute risk of IPD immediately following high concentrations of PM2.5, whereas times and locations with low prevalence of GPSC21 have an increased risk at later lag times (Figure 5C). In this dataset GPSC21 comprises serotype 19F (n=90) with one instance of 19A in 2005. Serotype 19F is targeted by PCV7 and is characterized in South Africa by an initial GPSC1-lineage (n=114) which decreased post-PCV when GPSC21–19F became predominant (Figure 5B)^22^. When we re-run the GPSC21 interaction models splitting by pre- and post-PCV period we see the effect persisting in the post-PCV period with poor model performance due to low-N in the pre-PCV period (Figure S14). While further investigation is required to understand the mechanism by which GPSC21–19F is causing invasive disease in the short-lag period following high levels of PM_2.5_, it is clear that there are some differences in environmental effects given the circulating lineage diversity.

## Discussion

In this study, we applied spatio-temporal Bayesian hierarchical models to investigate the relationship between PM_2.5_ exposure and the risk of IPD, while accounting for microbial diversity and key interventions (ART and PCV). In all presented models we accounted for interannual variability (replicated across provinces), seasonality, spatial autocorrelation, vaccination periods, and population density. We quantified the impact of ART and PCV on interannual variation in IPD, identified the effect of PM_*2.5*_ with variable lag-times of the effects across pneumococcal serotypes, age groups, and disease types, and found the greatest increased risk in older adults. We examined whether genomic lineages modified the effects of PM_2.5_ on IPD and found a higher short-term, acute (lag 0) effect of PM_2.5_ when GPSC21–19F was highly prevalent (RR = 1.04; 95% CI 1.01–1.06).

We identified significant protective effects of ART and PCV on IPD although ARTs exacerbated impact on post-2020 NVTs was unexpected. It may reflect the increase in adult IPD cases in that period, and a waning effect of PCV (the major decreases in VT serotypes occurred at earlier time points^22^). Overall the impact of ART on opportunistic infections cannot be understated^17,23–25^. Funding for programs which have enabled ART coverage to exceed 70% in South Africa are being diminished and there are shifting funding priorities despite the many important secondary benefits of ART. Cuts to the U.S. President Emergency Plan for AIDS Relief (PEPFAR) in 2025 has been estimated to disrupt daily ART to at least 222,000 people and could lead to an increase of 30–64,000 new HIV infections by 2028^26^. There will likely increase in IPD incidence with the loss of funding for ART.

Our study confirmed a positive relationship between PM_2.5_ and IPD with similar positive associations with PM_10_, and SO_2_. We focused the study on PM_2.5_ due to sources often being anthropogenic and amenable to action^27–29^. The impact of underlying pneumococcal population diversity on the effect of air pollution has not previously been explored^30^. Notably, when we stratified the models by serotype we found an immediate effect from PM_2.5_ on some specific serotypes including NVT serotype 8 and vaccine serotypes 4 and 23F but found a lagged effect for other serotypes. Different pneumococcal serotypes are known to vary in their invasive potential implying variability in disease mechanism^31^. The mechanism behind the effect of PM_2.5_ on IPD is likely both on the host side (e.g. increased susceptibility to colonization^11^, decreased mucociliary clearance^32^) and the bacterial side (e.g. increased transformation capacity^33^ and biofilm formation^34^)^13^. The immediate risk from PM_2.5_ on NVTs and specific VTs may imply that there is a fitness advantage for these serotypes in high pollution areas. The lagged effect among most VTs suggests that vaccines provide partial protection against immediate PM_2.5_-associated risk. The PM_2.5_ effects persist across all age groups independently. The most acute effect (immediate risk from PM_2.5_) is among those aged 15–64 years and the cumulative effect is greatest for those ≥65 years-old. Importantly, the risk for children <5 years-old is non-negligible. These associations have been previously described for adults and children, with highest risk for older adults^28,35,36^.

The pneumococcus is highly diverse and while the serotype data can summarize some of that diversity the immense recombination and dynamic population structure of the pneumococcus cannot be ignored. GPSCs have different carriage durations and demographic propensities^3,4^. It was not possible to directly stratify models including different GPSCs as an outcome, due to the high diversity (low-per-GPSC N), so we instead evaluated the interaction between environmental factors and GPSC prevalence. This allowed us to develop converging models adjusting for the underlying genomic population diversity.

GPSC21–19F is an emergent strain post-PCV in South Africa. The persistent serotype 19F (including PCV) is more frequently expressed by GPSC21 than the pre-PCV GPSC1–19F strain. Serotype 19F is especially prevalent among 5–18 year olds and behavioral differences may contribute to the acute effects of PM_2.5_ on GPSC21-IPD^22^. Direct experimental evidence linking lineage-specific mechanistic data on PM_2.5_-lineage data is currently lacking. Expanding genomic surveillance can support more mechanistic studies to investigate the specific biological interactions which may promote a PM_2.5_ fitness advantage for particular lineages.

The concentrations of PM_2.5_ and PM_10_ found in South Africa, especially the province of Gauteng, are extremely high. The strong positive association observed in this study is likely partially due to the high exposure concentration. The current threshold aim for PM_2.5_ in South Africa is 40 μg/m^3^ whereas the WHO threshold is 15 μg/m^3^. We found in the year 2019 that 14.8 cases might have been averted if the South African threshold was met (24.3 cases by meeting the WHO threshold). More stringent air pollution goals could directly mitigate IPD.

There is an inextricable, bidirectional link between PM_2.5_ and climate change in that particulate matter influences the Earth’s radiative balance and atmospheric chemistry, while changes in temperature, precipitation, and land use affect the formation, composition, and distribution of PM^37,38^. Further, burning of fossil fuels both increases air pollution and global temperatures. There is biological plausibility for air pollution directly impacting host susceptibility, and promoting a fitness advantage for particular microbial traits. Our findings show that high levels of air pollution create conditions that favor particular serotypes and lineages. We provide microbial diversity-aware estimates of the risk of IPD driven by air pollution, demonstrating that the risk is not uniform across human populations with variable effects on at-risk groups and disease types. These interactions should be studied further across different regions with varied climatic conditions and demographic structures. Mechanistic experiments could enhance our understanding of the particular biology leading to these interactions between microbial diversity, host susceptibility, and environment.

The time period, high spatial resolution, and extensive metadata of our study is an asset for robust space-time ecological modelling studies. We believe that this novel approach for including genomic data could be leveraged for other infectious diseases to incorporate microbial genomic data into environmental epidemiological models.

### Limitations

The reanalysis and observational datasets for PM_2.5_ are imperfect. As we demonstrate in our comparison, air quality reanalysis models do not correlate well with the observational data across all sites. The available observational data from South African Air Quality Information Services (SAAQIS) across this time period is sparse and limits our ability to select an ideal reanalysis product. The correlation between CAMS and the observations was better than MERRA-2 and other bias corrected datasets. The absence of reanalysis models which are bias corrected to this region, and lack of observational data points for many less-urban regions, highlights the limitations of addressing the environmental impact of high-burden infectious diseases in low-income settings, with uncorrected environmental data^39,40^. To improve understanding of the environment’s impact on high mortality infectious diseases efforts must be made to improve air pollution global reanalysis models^41^. The air quality standards we used as baseline values are the 24-hour standards set by NAAQS. These standards are higher than the health standards set by the WHO due to the high concentration of pollutants in some South African cities. As such, our relative risk estimates are calculated against threshold defined for the South African context but would be higher if compared to the WHO standards. We used weekly means rather than the 24-hour means for air quality metrics so these thresholds are lenient (e.g. if 1 day within a 7 day period has a 24 hour concentration exceeding the threshold but the other 6 days do not, the weekly mean concentration will likely be below the threshold).

Environmental reanalysis datasets should be expanded and refined across a wider range of geographies. This may require targeted investment in sustainable air quality monitoring networks, but the resulting evidence would elucidate air quality evolution and the impact on human health globally. Despite our inclusion of the entire GERMS-SA dataset across the time periods, sources of ascertainment bias include access to care, and correct processing and acquisition of specimens. Our data is from hospital location so that in cases of long-distance travel to hospitals cases may be attributed to a spatial grid different from the patient’s home location. While audits were conducted to capture unreported cases these excluded serotype data. While our study included a large proportion of genomic sequences these did not encompass the entire epidemiological dataset. The isolates selected for genomic sequencing were randomly selected according to specific age groups in each sequencing set (as described in the methods). By incorporating the genomic lineages as a modifying proportional interaction effect, and normalizing this by the total number of sequences in each space-time increment we try to limit these biases. We did not include carriage isolates in this analysis, so our results pertain only to IPD lineages and serotypes. We assume that the chosen lag window (8-weeks) for relevant covariates is sufficient to capture delayed effects and effects beyond this are assumed negligible.

### Conclusion

In summary, environmental factors influence IPD risk in South Africa with humidity and particulate matter posing the highest disease risk. The relationships we found are delayed and non-linear, and vary given the serotypes or lineage circulating. While there was a strong association between PM_2.5_ and IPD in all age groups independently, the highest risk is among those ≥65, and the most immediate risk is for those between 15 and 64. The lagged and variable effects indicate that the mechanism by which PM_2.5_ increases IPD risk varies across groups (e.g., ages, serotypes, immunity). This study provides strong evidence that diverse bacterial populations are not uniformly affected by the environment. IPD patterns are influenced by increasing urban populations and climate change which may have important implications for disentangling vaccine escape, vaccine implementation strategies, and for hospital capacity preparations in changing environments. Critically, it also highlights the need to meet the policy thresholds for PM_2.5_ to directly improve human health.

## Materials and Methods

### Data Processing

#### Pneumococcal Disease Datasets

##### Epidemiological Cases

The case counts of invasive pneumococcal disease (IPD) were provided by the National Institute for Communicable Diseases as part of national surveillance encompassing all nine provinces GERMS-SA. We included the entire dataset which comprised culture positive with viable isolates available for further analysis, culture positive with no viable isolates available, and culture negative with metadata results from PCR. Isolates were phenotypically characterised as has been previously described^22,42^. Finally, these included pneumococcal disease line list cases (N=59,017) between 2005–2023 collected from 531 hospitals across 52 districts in South Africa (Figure 1A–B). These excluded N=661 cases from the original dataset without hospital geolocation. Most cases were found in Gauteng with 24,303 cases. Northern Cape reported the least number of cases, with 1,067 (Figure S1C). Disease types included 32,639 bacteremia (55.3%), 21,167 cases of meningitis (35.9%), and 5,211 other IPD cases (8.8%), such as empyema, septic arthritis, endophthalmitis, peritonitis, or pericarditis. These were classified given specimen type whereby meningitis cases were isolated from cerebrospinal fluid specimens, bacteremia from blood specimens, and other invasive disease classes were from other typically sterile sites. The median age was 32 with 13% of cases reported in children <1 year old. There was missing age data for 4.3% of cases and serotype information was missing for 37.8% of cases (Figure 1C). We included data from 2005–2023 in concordance with the environmental datasets for the initial baseline epidemiological model testing. As the genomic dataset ran until 2020, the year marking the beginning of the SARS-CoV-2 pandemic (with a substantial decrease in IPD counts), we restricted the models to the period 2005–2019 (N=52,437) so as to overlap with epidemiological cases and exclude this case reduction. In the overall environmental epidemiology models we included all data and for the models stratifications by outcome we included all reported data for each outcome. The outcomes we assessed included different age groups, disease types, and serotypes.

##### Genomic Sequences

The genomes included in this study were sequenced as part of the Global Pneumococcal Sequencing project, GPS^43^ which is a global genomic survey of *S. pneumoniae*. Isolates were collected by the National Institute for Communicable Disease in South Africa from 2005–2020 (n=4,912) from 48 of the 52 districts. We only include genomes through the year 2019 (N=4,350). Isolates were randomly sampled using a previously described protocol whereby approximately 300 invasive-disease isolates from each year (2005–2014) were selected with a specific target age breakdown (50% from <3 year olds, 25% from 3–5 year olds, 25% from >5 year olds). In the second phase of sequencing, 100 disease isolates from 2005 to 2010 from children <5 years old were additionally, randomly sequenced. The sampling for 2015–2020 was similar however the sampling strategy was not adjusted for the reduction in IPD cases following PCV (thus included more >5 year-olds)^6,22,44^. Most sequences are from the districts with the highest population size (Figure S11). Assembly quality control parameters included a minimum average sequencing depth of 20× and an assembly length of 1.9–2.3 Mb. Sequences with more than 220 heterozygous SNP sites were excluded. The genomes were assembled using Spades assembly software^45^ and the gene annotations were made using Prokka^46^ PopPunk^47^ was used to assign each genome to a Global Pneumococcal Sequence Cluster (GPSC). We included each GPSC with an N>90 and included GPSC8 due to its public health relevance^22^. We included the proportion as the number of each GPSC over the total number of sequences per week per province (Figure S12).

##### Sociodemographic Data

The sociodemographic data was extracted from multiple sources. The population sizes per year per district were sourced from the 2023 Statistics South Africa projections^48,49^ From the same report we included per province ART coverage, HIV prevalence, life expectancy, growth rate, % children (<15) and the % elderly over time (60+). We also included the aging index and dependency ratio. These are defined by Statistics South Africa as the number over age 65 per 100 youths and the number <15 years old combined with the number 60+ over the number of working age population (15–64) respectively.^49^ The pneumococcal conjugate vaccine has a 2+1 schedule in South Africa and was first introduced with PCV7 in 2009 and PCV13 in 2011 (Table 1). PCV coverage for 1, 2, and 3 doses were included from the WHO Immunization Data report.^50^ Three doses is defined as the percentage of surviving infants who receive the 3rd dose of pneumococcal conjugate vaccines. We included the official coverage estimates as well as the WHO/UNICEF Estimates of National Coverage (WUENIC)^51^ (Figure S1D). The official estimates initially are based on surveys and other administrative messages, which, due to biases and inaccuracies, are adjusted by the national authorities to provide estimates of the most likely coverage.

#### Environmental Datasets

##### Meteorological Data

The environmental datasets spanned 2005–2023 inclusive. We included temperature, precipitation, relative, and absolute humidity metrics from the global reanalysis dataset ERA5-Land^52^ aggregated for weekly time points at the district level. This is gridded data with 0.1° degree resolution (accounting for the mean latitude of South Africa at −29° so each grid square is approximately 108km^2^). This resulted in a mean of 218 grid cells per district ranging from 15 in Johannesburg, Gauteng to 1180 in Namakwa, Northern Cape. Precipitation was initially retrieved as hourly average meters (m) per second precipitation loss rate (prlr). We converted it to daily millimeters (mm).

We calculated the Standardised Precipitation Index (SPI) and Standardised Precipitation and Evapotranspiration Index (SPEI) at 1-month, 3-month, 6-month, and 12-month time periods using the above mentioned precipitation and temperature data from ERA5-Land. The packages used for this calculation were SPEI^53^, ClimProjDiags^54^, and CSTools^55^.

Absolute humidity (absh) is defined as the mass of water vapour in a volume of air and the units are g/m^3^. Relative humidity (hurs) is the water vapour present in the air relative to the saturated air and defined as the partial pressure of water vapour as a percentage of the saturation pressure (%)(Figure 1D).

##### Air Pollution Data

The air pollution datasets spanned 2005–2023 inclusive. To determine which estimates of air pollution to include we compared air pollution estimates obtained from Copernicus Atmosphere Monitoring Service (CAMS) global reanalysis dataset, and NASA’s MERRA-2^56,57^ We performed an assessment of the correlation between the gridded air quality reanalysis models against observational data made available by the South African Air Quality Information Services (SAAQIS)^58^. PM_2.5_ and PM_10_ are not directly included in MERRA-2, so they were computed using methods detailed in the MERRA-2 documentation^59^(Figure 1D)(Table 2).

We included the MERRA-2 product from NASA for the entire time period and also included the bias corrected MERRA-2 product from 2020–2023^60^ The CAMS global datasets are at approximately an 80km^2^ resolution while the MERRA-2 datasets are at a 50km^2^ spatial resolution. We also included a gapless 1km gridded machine learning model from Wei *et al.*, 2023^61^

We extracted observational data from air quality monitoring stations provided by SAAQIS in South Africa. We calculated a mean absolute error, root mean square error, and Spearman’s correlation between the reanalysis products and the mean across observations in the district of Gert Sibande, Mpumalanga, Gauteng province, and KwaZulu-Natal province (Figure S15A). These were the locations with the most consistent observations across the time period available in SAAQIS. We performed a formal comparison of the datasets against observational data and found that CAMS was the best performing reanalysis model (Figure S15B). CAMS performed best despite the poor performance in the Gert Sibande region where none of the products performed well, either due to the observations being flawed, or a bias across all reanalysis products impacting this region. We included the air quality reanalysis data from CAMS in our study (Table 3).

The R package startR was used to retrieve and reformat all multidimensional environmental datasets^62^.

### Epidemiological Model Specification

#### Bayesian hierarchical mixed models using integrated nested laplace approximation

##### Statistical Analysis

All statistical analysis and visualizations were conducted using R version 4.4.2^63^ with key packages including R-INLA^64^, dlnm^65^, dplyr^66^, sf^67^, and vegan^68^.

##### Base Model Evaluation

We modeled weekly pneumococcal disease counts in South Africa between 2005–2023 (19 years) by specifying a Bayesian hierarchical mixed model using pneumococcal disease case counts at the district (N=52) or province (N=9) level as the response variable. To account for overdispersion, we assumed that case counts follow a negative binomial distribution such that:

$${\text{y}}_{\text{s},\text{t}}|\mu_{\text{s},\text{t}}\sim \text{NegBin}\left(\mu_{\text{s},\text{t}},\kappa \right)$$

$$\text{log}\left(\mu_{\text{s},\text{t}}\right)=\text{log}\left({\text{p}}_{\text{s},\text{a}\left(\text{t}\right)}\right)+\text{log}\left(\rho_{\text{s},\text{t}}\right)$$

where μ_s,t_ is the expected number of cases and κ is the overdispersion parameter. The distribution mean, μ_s,t_, is defined as the product of the population p_s,a(t)_ (per 100,000 people) in a given spatial unit, s, (district or province) and year a(t), and the disease incidence ρ_s,t_.

We tested increasingly complex baseline models ultimately selecting M12 as our baseline model (Table 4)(Table S2). We evaluated the model performance defining the response variable both at district and province level for weekly and monthly case counts and across various random and fixed effect combinations. We proceeded with the weekly models due to their relevance for respiratory disease effects (Table S2).

Baseline model, M12, included an intercept α as well as spatiotemporal random effects accounting for unknown and unmeasured monthly effects (calendar months January (1) to December (12)), δ_m(t)_, and interannual effect replicated by province (n=9 provinces) γ_a(t,pr)_. This model also included spatial effects with a Besag-York-Mollié (BYM-2)^69^ including both structured (*u*_*s*_) and unstructured effects (*v*_*s*_).

We included a single categorical covariate with three-phases to account for decreasing case counts after the implementation of the pneumococcal conjugate vaccine (PCV): pre-PCV (years before 2009), PCV7 (2009–2010 inclusive), and PCV13 (2011 onwards). This accounted for a non-linear effect from vaccination. We also included district level human population density (*pd*) as a covariate due to its particular relevance as a confounding variable with particulate matter. In the province level model *s* = *pr*.


$$\text{log}\left(\rho_{\text{t},\text{s}}\right)=\alpha +\delta_{\text{m}\left(\text{t}\right)}+\gamma_{\text{a}\left(\text{t},\text{pr}\right)}+{\text{u}}_{\text{s}}+{\text{v}}_{\text{s}}+\text{pc}{\text{v}}_{\text{periods}}+\text{p}{\text{d}}_{\text{s},\text{a}\left(\text{t}\right)}$$

We evaluated models with different random effect formulations and compared them to the intercept only model. We found an increasing goodness-of-fit with increasing complexity (Table S2). We selected our baseline model due to the balance of complex random effects and goodness-of-fit.

#### Impact of antiretroviral therapy and pneumococcal vaccine on interannual variation

To assess the impact of the uptake of ART (treatment for HIV) across South Africa compared to the implementation of the vaccine we evaluated models with them included independently and together. We tested different models including the ART and PCV covariates with different formulations both together and independently. These included: i) PCV as a categorical variable with 2 categories pre-PCV7 (2005–2008), and post-PCV (2009–2023) (M10); ii) PCV as a single categorical covariate with 3 categories for three vaccine periods (pre–PCV7, PCV7, and PCV13) (M11); iii) PCV coverage with one value (% coverage) nationally per year (M13); iv) ART coverage with one value (% coverage) nationally per year (M14); v) ART coverage with one value (% coverage), per province, per year (M15); vi) and a model including ART % coverage per province/per year and a categorical covariate covering the 3 vaccination periods (M16) (Table 4). These were all run with and without a replication of the interannual effect across provinces (Table S4). We also ran different models for different types of IPD to assess the impact of these perturbations across different groups. These included children ≤5 years old, adults between 15 and 64 years old, adults 65 and above, IPD from only NVTs and from only VTs. We also ran these models including disease from 2005–2019 (excluding the post-COVID-19 period), and from 2005–2023 (Table S4).

#### Distributed lag non-linear models to estimate disease risk with environmental factors

##### Overall Models

To account for lag times from the exposure to disease outcome we specified distributed lag non-linear models (DLNMs) to estimate risk of pneumococcal disease incidence for different environmental factors and lag times^31,35,4^. To explore the data, we correlated lag times from exposure to disease outcome using a Spearman correlation (Figure S16). We included cases from 2005–2019 to match our genomic sequence models. We ran weekly models at both province (adm1) and district (adm2) level. We evaluated 6, 8, and 12 week lag times across the variables and administrative regions finding that 8 weeks of lags provided the best model fit. We generated the DLNM crossbasis with 8 weeks of lag with 2 degrees of freedom for the lag knots except for relative humidity for which we included 3 degrees of freedom to capture the complex non-linearity. For each variable we included equally distributed knots at the 33rd and 67th quantile except for precipitation for which we included 2 equally spaced knots on a log scale of the precipitation distribution. We centered and scaled each environmental variable. We evaluated the Pearson correlation between the environmental variables. We also tested models including the province of Gauteng alone due to it being the most populous, highest incidence of IPD, and highest maximum concentrations of PM_2.5_, PM_10_, and SO_2_ (Figure S4)(Figure S5).

For the best models (relative humidity, absolute humidity, PM_2.5_ and PM_10_) with R^2^>0.6 we evaluated the inclusion of additional environmental covariates (maximum temperature, PM_2.5_, PM_10_, SO_2_) as linear terms. We also tested the addition of relative humidity and absolute humidity as non-linear effects using random walk 2 models with 5 cuts. Because the model uses a log-link, additive terms in the linear predictor correspond to multiplicative effects at the response level (i.e. disease incidence rates). We estimated the exposure-lag-response association using the crosspred function. For air quality metrics we centered at the 24-hour National Ambient Air Quality Standards (NAAQS) for South Africa (PM_2.5_ = 40, PM_10_ = 75, and SO_2_ = 125 μg/m^3^). We utilized the 24 hour exceedance threshold rather than the annual as we are examining an acute effect (IPD) (Table 5). For the rest we centered this estimation at the median of the variable distribution (Table S5).

##### Stratification by age, disease, and vaccine status

To identify differences in risk across different stratification groups we changed our outcome from IPD overall to disease by age group, disease outcome, vaccine status (whether cases were serotypes which were included in the PCV7 vaccine, PCV13 vaccine, or were non-vaccine type), and by specific serotype. For these we included the M12 baseline model (Table 4), but we changed the outcome to disease incidence among each of these groups per 100,000 population. The models tested included i) age ≤5 years old, ii) age 15–64 years old, iii) age ≥65, iv) bacteremia, v) meningitis, vii) other invasive disease types. We also included serotype stratifications by vii) NVT, viii) PCV7, and ix) PCV13. Models 11–28 included stratification by IPD from particular serotypes (Table S1). We initially included ages 6 to 14 and found no effect across any lag-time or cumulatively with large credible intervals so exclude this group in the main results.

To estimate the relative risk ratio between each subgroup (serotype, disease type, age group) compared to the overall population we crosscut the relative risk from the exposure-response curve at 50 μg/m^3^ concentration for each. We then included the relative risk for each subgroup in turn as the numerator compared to the relative risk from the overall model to estimate the increased (>1) or decreased (<1) risk of IPD, in each subgroup, from PM_2.5_ compared to the risk for pneumococcal disease overall (Figure 4A).

#### Distributed lag non-linear models interacting with lineage prevalence

To assess the impact of underlying lineage diversity on the environmental effects we utilized the same baseline model (M12: seasonal, spatial, and province-replicated-interannual random effects, together with vaccination periods and population density)(Table 4). We then included an interaction between the exposure crossbasis and a modifying variable representing GPSC-specific proportions per total number sequenced each week. We only included a province level spatial effect for these interaction models. To ensure model interpretability, we scaled and centered this variable (Table S6). We only included space-time units in which each modifying variable is non-zero.

We evaluated the exposure–response relationship at three representative values of the effect modifier, corresponding to the 0.1, 0.5, and 0.8 quantiles of the centered distribution of the modifying variable (excluding zeros). For each quantile value *z*, we derived level-specific coefficient vectors and corresponding variance–covariance matrices that reflect the combined main and interaction effects.

To account for uncertainty in the interaction effects across different levels of the modifying variable, we computed level-specific coefficient vectors and their corresponding variance-covariance matrices. For a given value z of the centered interaction variable, the coefficient vector was calculated as:

$$\beta_{z}=\beta_{0}+z\cdot \beta_{int}$$

and the associated variance-covariance matrix as:

$$V_{z}=V_{0}+z^{2}\cdot V_{int}+2z\cdot V_{\text{cross}}$$

where β_0_ and *V*_0_ are the estimated coefficients and variance-covariance matrix for the main (non-interaction) effect, β_*int*_ and V_*int*_ are those for the interaction term, and *V*_*cross*_ represents the covariance between main and interaction coefficients. These were then passed to the crosspred() function of the dlnm package to generate estimates and confidence intervals at each value of *z* (Table S6).

We performed a sensitivity analysis whereby for GPSC pairs, which share the same dominant serotypes, we split the dataset into the pre- and post-vaccine period to ensure the effect was not just driven by serotype replacement in the time series but by some underlying evolutionary process (Figure S14).

To estimate goodness-of-fit for each model we calculated the Watanabe-Akaike information criterion (WAIC)^71^and the mean absolute error (MAE). We also calculated the sum of the log10 conditional predictive ordinate (CPO) for each observation to evaluate the model’s out-of-sample predictive performance. We additionally computed a pseudo-R^2^, defined as the proportionate reduction in deviance compared to an intercept-only model^72^ This measured the ability of each model to account for variation in disease incidence (0–1) whereby a model with R^2^ = 1 would indicate a model accounting for all of the variation in the data. This helped evaluate the improvement in model fit achieved by including additional covariates.

## Supplementary Material

Supplement 1

1

## Figures and Tables

**Table 1.** Vaccine implementation year, schedule, and serotypes in South Africa. 2+1 indicates a 2+1 schedule whereby children receive the first two doses at 6 and 14 weeks of age and then a booster dose at 9 months.

Vaccine (introduction year in South Africa and schedule)	Serotypes
PCV7 (2009; 2+1)	4, 6B, 9V, 14, 18C, 19F, 23F
PCV13 (2011, 2+1 )	4, 6B, 9V, 14, 18C, 19F, 23F, 1, 3, 5, 6A, 7F, 19A

**Table 2.** Description of air quality reanalysis products. Calculations to adjust each air-quality reanalysis product to μg/m^3^ detailing the mass used for PM_2.5_, PM_10_, O_3_, and SO_2_. #

Variable	CAMS (50km^2^)		MERRA (80km^2^)		MERRA_BiasFix (80km^2^)	
Adjustment	Initial	Adjust^#^ to μg/m^3^	Initial	Adjust^#^ to μg/m^3^	Initial	Adjust to μg/m^3^
PM_2.5_	kg m ^−3^	*10^9^	kg m ^−3^	*10^9^	kg m ^−3^	*10^9^
PM_10_	kg m ^−3^	*10^9^	kg m ^−3^	*10^9^	-	-
O_3_	kg kg ^−1^	*2.14*10^9^	kg kg ^−1^	*2.14*10^9^	-	-
SO_2_	kg kg ^−1^	*2.63*10^9^	kg kg ^−1^	*2.63*10^9^	-	-

# air density volume included as 1.23, O_3_ density as 2.14 and SO_2_ as 2.63

**Table 3.** Correlations between observational station data and each air quality analysis product. Relationship between reanalysis products from Copernicus (CAMS), MERRA-2 from NASA, MERRA-2 with a bias adjustment and a machine learning gapless product at 1km grid squares from Wei et al. 2023. We include a spearman’s correlation, mean absolute error (μg/m^3^) and the root mean squared error (μg/m^3^).

	Gauteng				KwaZulu-Natal				Gert Sibande, Mpumalanga			
Statistic	CAMS	MERRA-2	MERRA-2-adjusted	ML Gapless 1km	CAMS	MERRA-2	MERRA-2-adjusted	ML Gapless 1km	CAMS	MERRA-2	MERRA-2-adjusted	ML Gapless 1km
Correlation	0.88	0.49	0.58	0.82	0.58	0.52	0.17	0.08	0.08	0.30	−0.21	0.27
MAE	43.06	74.79	62.05	55.52	32.49	52.98	50.74	48.15	74.55	92.95	83.27	83.40
RMSE	58.46	81.02	67.47	61.40	42.34	59.69	57.50	55.69	105.45	119.56	113.56	111.66

**Table 4.** Model formulations with increasing complexity to determine baseline model at a weekly temporal resolution and including years 2005–2019. WAIC = Watanabe-Akaike Information Criterion, MAE = Mean Absolute Error, CPO = log10 conditional predictive ordinate, and R^2^ likelihood ratio compared to the intercept model.

Model	Formula	WAIC	MAE	CPO	R^2^
M0	${\text{log}}_{\left(\rho \text{t},\text{s}\right)}=\alpha$	99687.81	1.18	1.23	0
M1	${\text{log}}_{\left(\rho \text{t},\text{s}\right)}=\alpha +\delta_{\text{m}(\text{t})}+\gamma_{\text{a}(\text{t})}$	97301.75	1.14	1.2	0.06
M2	${\text{log}}_{\left(\rho \text{t},\text{s}\right)}=\alpha +\delta_{\text{m}(\text{t})}+{\text{u}}_{\text{s}}+{\text{v}}_{\text{s}}$	84082.5	0.9	1.04	0.32
M3	${\text{log}}_{\left(\rho \text{t},\text{s}\right)}=\alpha +\gamma_{\text{a}(\text{t})}+{\text{u}}_{\text{s}}+{\text{v}}_{\text{s}}$	82439.36	0.8	1.02	0.35
M4	${\text{log}}_{\left(\rho \text{t},\text{s}\right)}=\alpha +\delta_{\text{m}(\text{t})}+\gamma_{\text{a}(\text{t})}+{\text{u}}_{\text{s}}+{\text{v}}_{\text{s}}$	80883	0.76	1	0.37
M5	${\text{log}}_{\left(\rho \text{t},\text{s}\right)}=\alpha +\delta_{\text{m}(\text{t})}+\gamma_{\text{a}(\text{t})}+{\text{u}}_{\text{s}}+{\text{v}}_{\text{s}}+{\text{pcv}}_{2009\text{vaccine}}$	80882.95	0.76	1	0.37
M6	${\text{log}}_{\left(\rho \text{t},\text{s}\right)}=\alpha +\delta_{\text{m}(\text{t})}+\gamma_{\text{a}(\text{t})}+{\text{u}}_{\text{s}}+{\text{v}}_{\text{s}}+{\text{pcv}}_{\text{periods}}$	80882.89	0.76	1	0.37
M7	${\text{log}}_{\left(\rho \text{t},\text{s}\right)}=\alpha +\delta_{\text{m}(\text{t})}+\gamma_{\text{a}(\text{t})}+{\text{u}}_{\text{s}}+{\text{v}}_{\text{s}}+{\text{pcv}}_{\text{periods}}+\text{covid}$	80883.01	0.76	1	0.37
M8	${\text{log}}_{\left(\rho \text{t},\text{s}\right)}=\alpha +\delta_{\text{m}(\text{t},\text{pr})}+\gamma_{\text{a}(\text{t})}+{\text{u}}_{\text{s}}+{\text{v}}_{\text{s}}+{\text{pcv}}_{\text{periods}}$	80794.05	0.75	1	0.38
M9	${\text{log}}_{\left(\rho \text{t},\text{s}\right)}=\alpha +\delta_{\text{m}(\text{t})}+\gamma_{\text{a}(\text{t},\text{pr})}+{\text{u}}_{\text{s}}+{\text{v}}_{\text{s}}$	78943.71	0.7	0.97	0.41
M10	${\text{log}}_{\left(\rho \text{t},\text{s}\right)}=\alpha +\delta_{\text{m}(\text{t})}+\gamma_{\text{a}(\text{t},\text{pr})}+{\text{u}}_{\text{s}}+{\text{v}}_{\text{s}}+{\text{pcv}}_{2009\text{vaccine}}$	78942.09	0.7	0.97	0.41
M11	${\text{log}}_{\left(\rho \text{t},\text{s}\right)}=\alpha +\delta_{\text{m}(\text{t})}+\gamma_{\text{a}(\text{t},\text{pr})}+{\text{u}}_{\text{s}}+{\text{v}}_{\text{s}}+{\text{pcv}}_{\text{periods}}$	78939.5	0.7	0.97	0.41
M12	${\text{log}}_{\left(\rho \text{t},\text{s}\right)}=\alpha +\delta_{\text{m}(\text{t})}+\gamma_{\text{a}(\text{t},\text{pr})}+{\text{u}}_{\text{s}}+{\text{v}}_{\text{s}}+{\text{pcv}}_{\text{periods}}+{\text{pd}}_{\text{s},\text{a}(\text{t})}$	78766.03	0.7	0.97	0.41
M13	${\text{log}}_{\left(\rho \text{t},\text{s}\right)}=\alpha +\delta_{\text{m}(\text{t})}+\gamma_{\text{a}(\text{t},\text{pr})}+{\text{u}}_{\text{s}}+{\text{v}}_{\text{s}}+{\text{pcv}}_{\text{national}}$	78940.44	0.70	0.97	0.41
M14	${\text{log}}_{\left(\rho \text{t},\text{s}\right)}=\alpha +\delta_{\text{m}(\text{t})}+\gamma_{\text{a}(\text{t},\text{pr})}+{\text{u}}_{\text{s}}+{\text{v}}_{\text{s}}+{\text{art}}_{\text{national}}$	78939.15	0.70	0.97	0.41
M15	${\text{log}}_{\left(\rho \text{t},\text{s}\right)}=\alpha +\delta_{\text{m}(\text{t})}+\gamma_{\text{a}(\text{t},\text{pr})}+{\text{u}}_{\text{s}}+{\text{v}}_{\text{s}}+{\text{art}}_{\text{national}}$	78938.82	0.70	0.97	0.41
M16	${\text{log}}_{\left(\rho \text{t},\text{s}\right)}=\alpha +\delta_{\text{m}(\text{t})}+\gamma_{\text{a}(\text{t},\text{pr})}+{\text{u}}_{\text{s}}+{\text{v}}_{\text{s}}+{\text{art}}_{\text{national}}+{\text{pcv}}_{\text{periods}}$	78938.55	0.70	0.97	0.41

**Table 5.** Air quality standards. For the years 2016–2029 as set by the Environmental Management Air Quality Act 39 in South Africa and the standards set by the WHO in 2021.

Air Quality Component	SA 24-hour	WHO 24-hour
**PM_2.5_**	40	15
**PM_10_**	75	45
**SO_2_**	125	40

* Standards set by the South African environmental management air quality act for 2016–2029^21^

## Data Availability

All code is available in the GitHub repository (https://github.com/sophbel/env_sa_manuscript).
