# Supplementary material for: Microbial diversity mediates the impact of air pollution on pneumococcal disease risk
Source: medRxiv. 2025 Dec 11:2025.12.10.25341877. Preprint. [Version 1] doi: 10.64898/2025.12.10.25341877 (PMC12706618; doi:10.64898/2025.12.10.25341877)
Supplement: 1 [file NIHPP2025.12.10.25341877V1-supplement-1.pdf]

medRxiv preprint doi: https://doi.org/10.64898/2025.12.10.25341877; this version posted December 11, 2025. The copyright holder for this preprint (which was not certified by peer review) is the author/funder, who has granted medRxiv a license to display the preprint in perpetuity.

It is made available under a CC-BY 4.0 International license .

Supplementary Materials

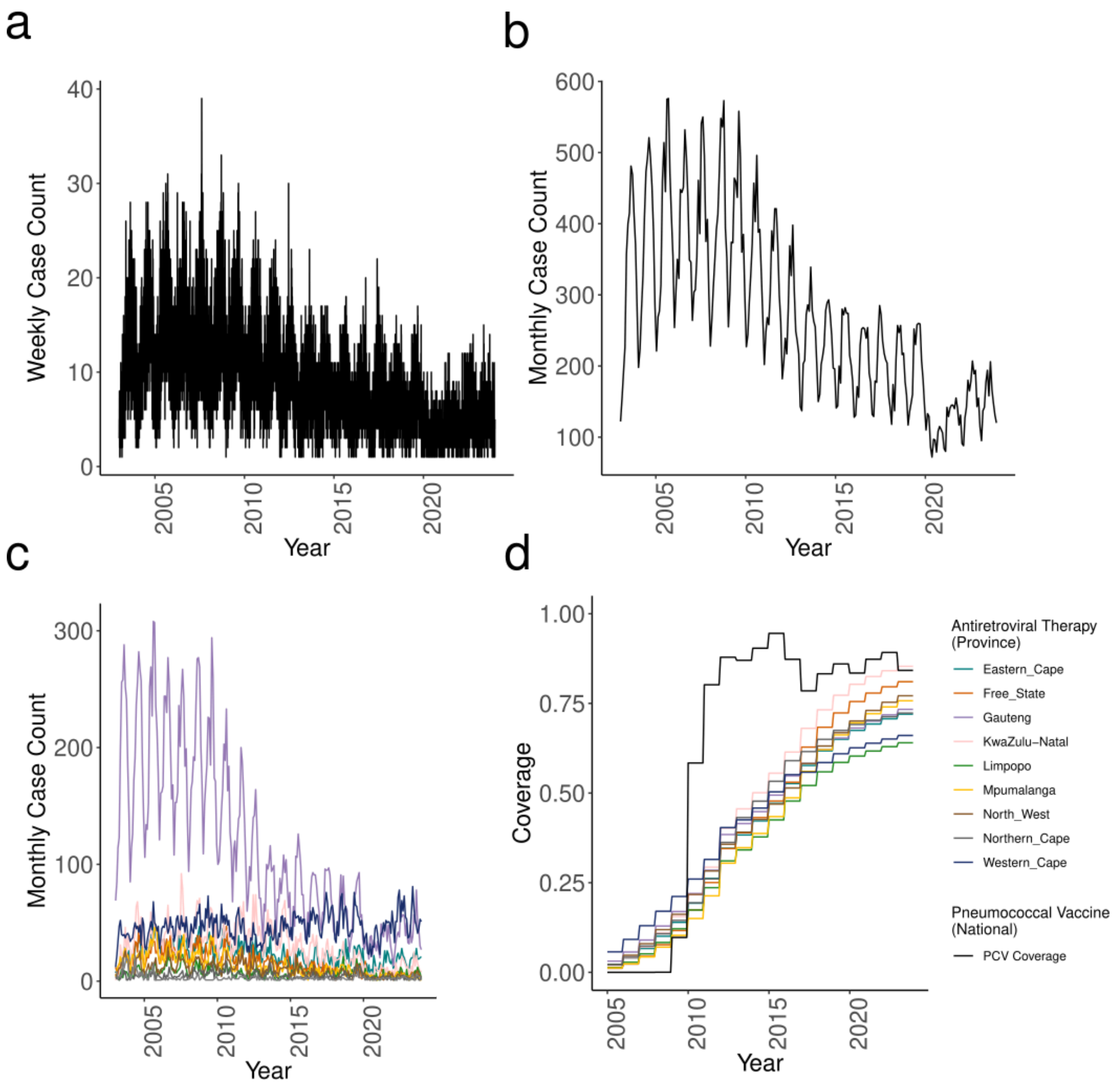

Figure S1. Pneumococcal case counts (a-c) and intervention coverage (d) (a) Weekly (a) and Monthly (b) pneumococcal disease case counts. (b) Disease counts monthly colored by province. (c) pneumococcal conjugate vaccine (PCV) coverage nationally and antiretroviral therapy coverage by province over time.

medRxiv preprint doi: https://doi.org/10.64898/2025.12.10.25341877; this version posted December 11, 2025. The copyright holder for this preprint (which was not certified by peer review) is the author/funder, who has granted medRxiv a license to display the preprint in perpetuity.

It is made available under a CC-BY 4.0 International license .

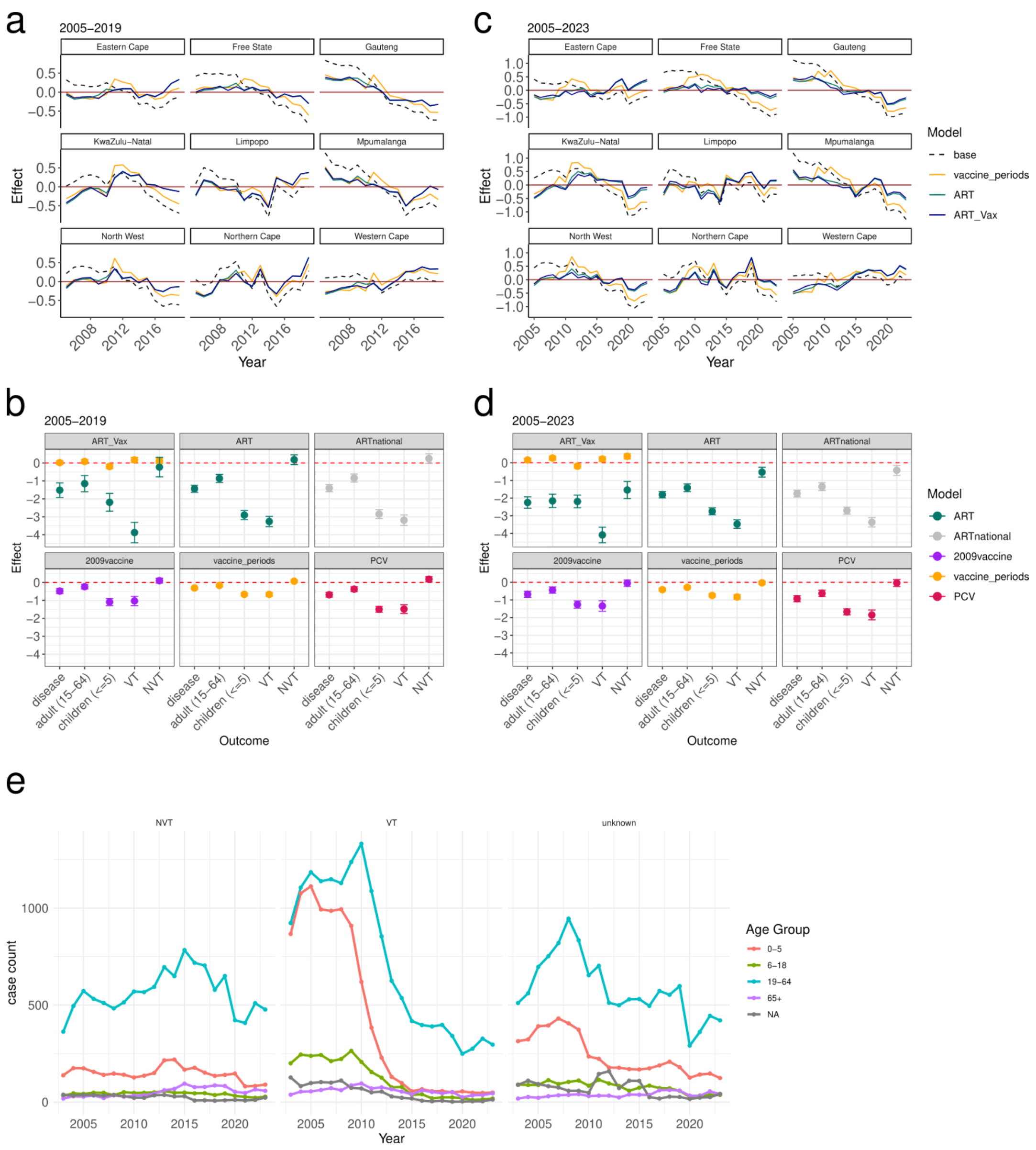

Figure S2. Effects from implementation of ART and vaccine included in the models with years (a-b) 2005-2019 and (c-d) 2005-2023. (a) and (c) show the interannual random effects for the base model with no perturbations (black, dashed), for a model with vaccination periods 2009 and 2011 (yellow), for ART coverage per province (green), and for a model including both vaccination periods and ART coverage per province (blue). (b) and (d) include a model with ART_Vax: both ART coverage per province (green) and vaccination periods (yellow), ART: a model with ART coverage per province (green), ARTnational: ART coverage nationally (grey), 2009vaccine: a model with a categorical variable indicating pre-PCV7 (2005-2008) and post-PCV (2009-2023) (purple), vaccine_periods: a categorical covariate indicating the pre-PCV, PCV7 (2009-2010), and PCV13 (2011-2023) periods (yellow), and a model with PCV coverage nationally (pink). These each were run for outcomes of all IPD, IPD in adults 15-64, children ≤5, VT serotypes, and NVT serotypes independently. (e) shows the case count across years and by age group including 0-5 (pink), 6-18 (green), 18-64 (blue), 65+ (purple), and absent age data (NA) (grey) for disease from NVTs (left), VTs (middle), and unknown serotype (right).

medRxiv preprint doi: https://doi.org/10.64898/2025.12.10.25341877; this version posted December 11, 2025. The copyright holder for this preprint (which was not certified by peer review) is the author/funder, who has granted medRxiv a license to display the preprint in perpetuity.

It is made available under a CC-BY 4.0 International license .

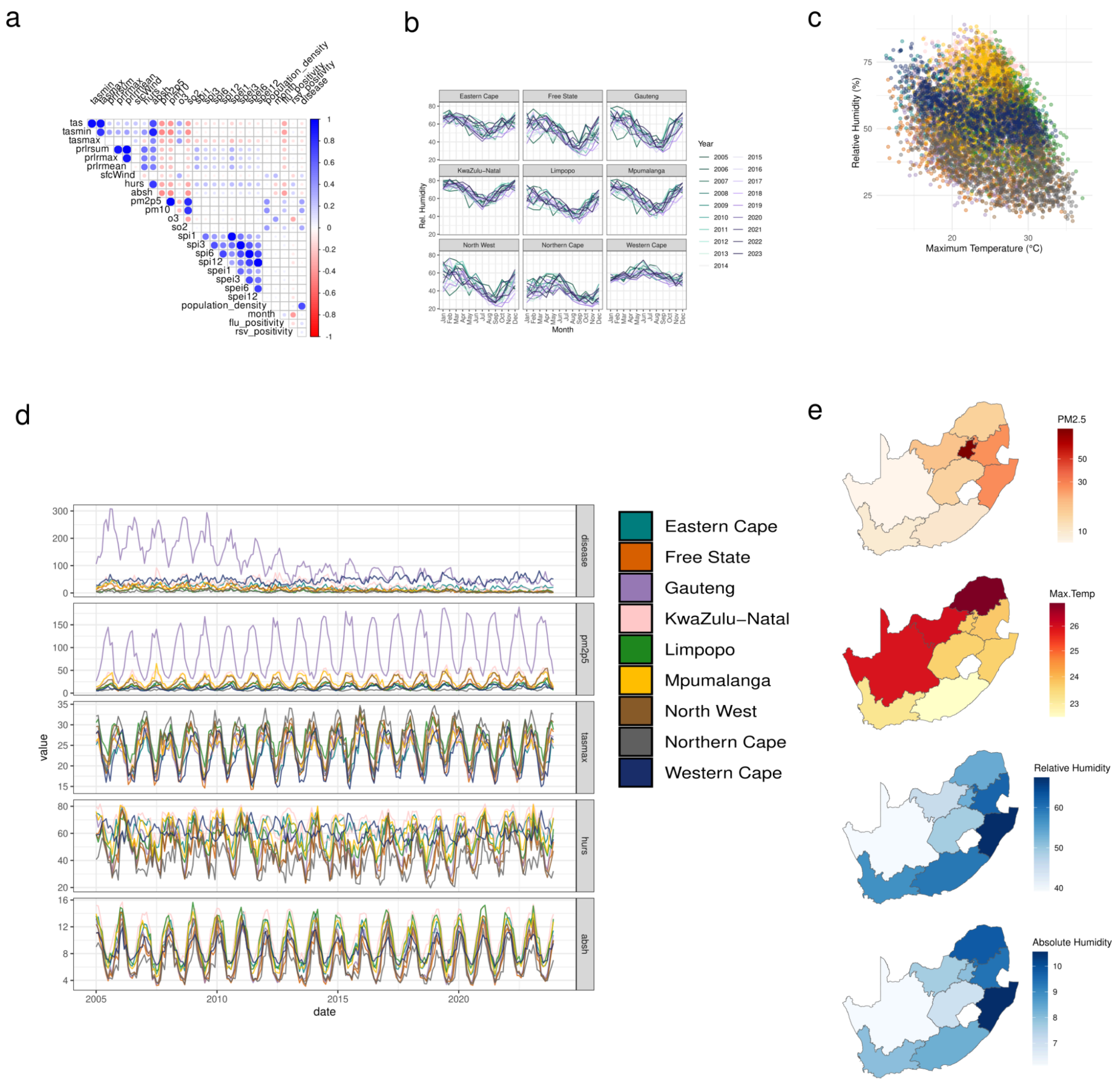

Figure S3. Summaries of included environmental variables. (a) correlation plot with spearman correlation between each of the external variables included. (b) relative humidity across months colored by the years of the study faceted by province. (c) Correlation between maximum temperature and relative humidity colored by province (same legend as d). (d) time series of pneumococcal disease case counts and (e) mean of each environmental variable per the 9 provinces of South Africa (d-e) $PM_{2.5}$ concentration ($\mu g/m^3$), maximum temperature (celsius), relative humidity (%), and absolute humidity (g/m^3 water) across 19 years colored by province.

medRxiv preprint doi: https://doi.org/10.64898/2025.12.10.25341877; this version posted December 11, 2025. The copyright holder for this preprint (which was not certified by peer review) is the author/funder, who has granted medRxiv a license to display the preprint in perpetuity.
It is made available under a CC-BY 4.0 International license .

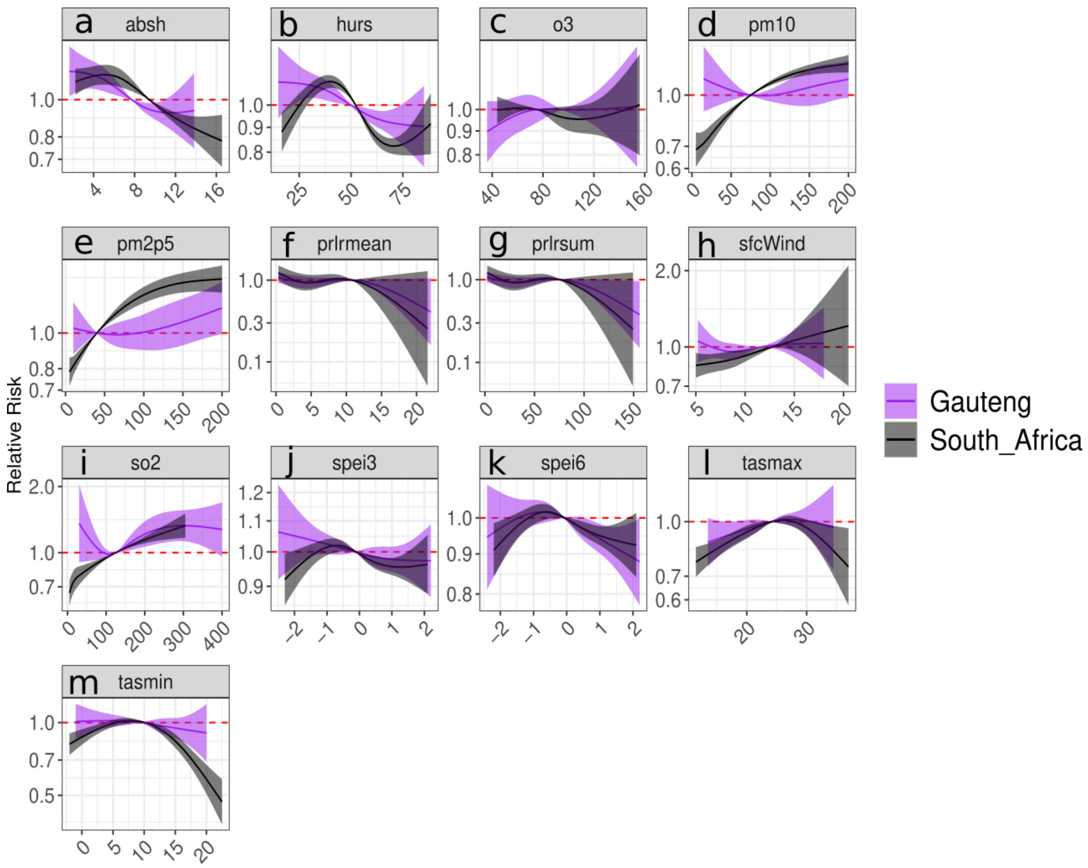

Figure S4. Cumulative exposure-response curves across 8 weeks of lags for the relative risk (y-axis) from each environmental variable (x-axis) in univariable DLNM models for Gauteng alone (purple) and South Africa overall (black). These models are fit to weekly district level case data with the baseline model random and fixed effects (seasonal, spatial, interannual, 3 vaccination period, and population density). (a) absolute humidity (absh) in g/m^3 water (b) percent relative humidity (c) ozone (o3) (d) particulate matter <10µm (pm10) (e) particulate matter <2.5µm (pm2p5) (f) mean precipitation millimeters (g) cumulative precipitation in millimeters (h) mean wind speed in km/hour (i) sulfur dioxide (so2) (j) drought index including evapotranspiration across previous 3 months (k) drought index including evapotranspiration across previous 6 months (l) maximum temperature (celsius) (m) minimum temperature (celsius).

medRxiv preprint doi: https://doi.org/10.64898/2025.12.10.25341877; this version posted December 11, 2025. The copyright holder for this preprint (which was not certified by peer review) is the author/funder, who has granted medRxiv a license to display the preprint in perpetuity.

It is made available under a CC-BY 4.0 International license .

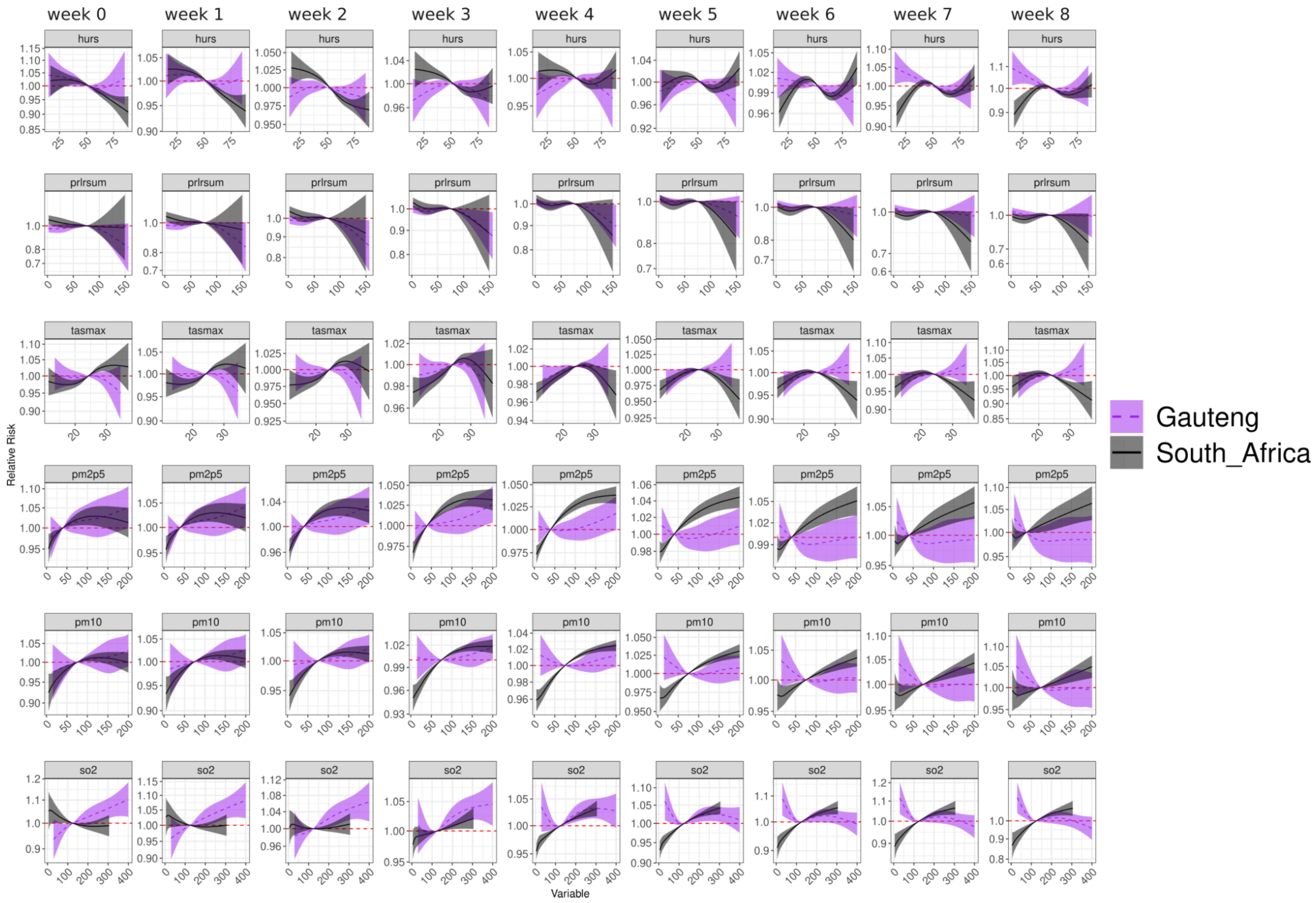

Figure S5. Exposure-response curves across 8 weeks of lags for the relative risk (y-axis) from each environmental variable (x-axis) in univariable DLNM models for Gauteng alone (purple) and South Africa overall (black). These models are fit to weekly district level case data with the baseline model random and fixed effects (seasonal, spatial, interannual, 3 vaccination period, and population density). The metric for each environmental variable is on the x-axis and the relative risk is on the y-axis. From top to bottom these include % relative humidity (hurs), cumulative precipitation (prlrsum) in millimeters, maximum temperature in celsius (tasmax), $PM_{2.5}$ (pm2p5), PM_{10}, and SO_2 concentrations in μg/m^3.

medRxiv preprint doi: https://doi.org/10.64898/2025.12.10.25341877; this version posted December 11, 2025. The copyright holder for this preprint (which was not certified by peer review) is the author/funder, who has granted medRxiv a license to display the preprint in perpetuity.

It is made available under a CC-BY 4.0 International license .

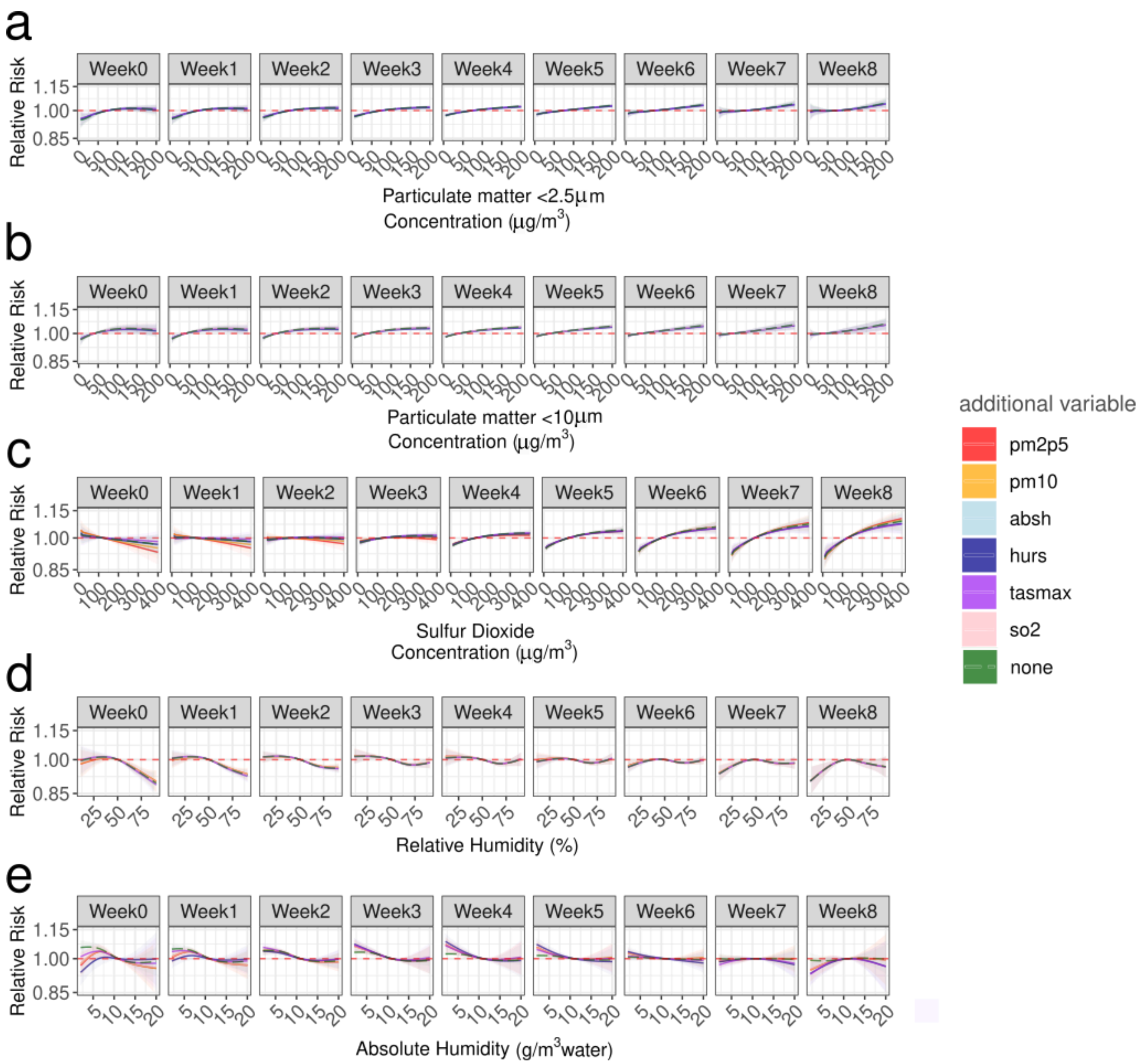

Figure S6. Relative risk across 8-weeks of lags for $PM_{2.5}$, PM_{10} SO_2, relative, and absolute humidity with additional multiplicative variables. In the DLNM the humidity variable cross basis has 3-degrees of freedom (df), while the pollution variables have 2. The additional multiplicative effects are included as fixed effects except the humidity variables which are non-linear with 5 cuts. Each plot includes the exposure-response curve for each lag-week colored by the multiplicative variable for (a) $PM_{2.5}$ (μg/m^3) (models for none, absh, hurs, tasmax, SO_2) (b) PM_{10} (μg/m^3)(models for none, absh, hurs, maximum temperature (tasmax), so2), (c) SO_2 (μg/m^3)(models for none, $PM_{2.5}$, $PM_{10,}$ absh, hurs, tasmax) (d) % relative humidity (hurs) (models for none, $PM_{2.5}$, PM_{10}, SO_2, tasmax) , and (e) absolute humidity (absh) (g/m^3)(models for none, $PM_{2.5}$, PM_{10}, $SO_{2,}$ tasmax).

medRxiv preprint doi: https://doi.org/10.64898/2025.12.10.25341877; this version posted December 11, 2025. The copyright holder for this preprint (which was not certified by peer review) is the author/funder, who has granted medRxiv a license to display the preprint in perpetuity.

It is made available under a CC-BY 4.0 International license .

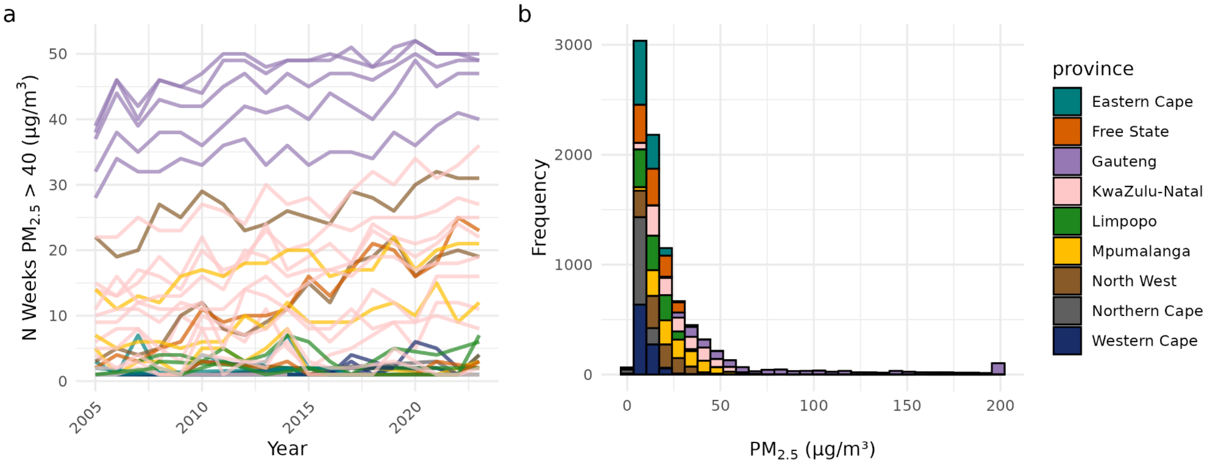

Figure S7. Particulate matter <2.5 µm distributions (a) weeks with >40 µg/m^3 (the NAAQS threshold for $PM_{2.5}$) per year and district colored by province across the time period. The grouping is by district whereby in Western Cape (dark blue) and Northern Cape (grey) have no weeks exceeding the threshold. Eastern Cape (blue) and Limpopo (green) only have districts with <10 weeks (blue), Free State includes Fezile Dabi (orange), Gauteng includes the City of Johannesburg, City of Tschwane, Ekurhuleni, Sedibeng, and West Rand (purple), KwaZulu-Natal includes Amajuba, eThekwini, Harry Gwala, iLembe, Ugu, uMgungundlovu, uMzinyathi, and uThukela (pink), Mpumalanga includes Gert Sibande and Nkangala with >10 weeks exceeding threshold (yellow), North West includes Bojanala Platinum and Dr Kenneth Kaunda with > 10 weeks (brown) (b) histogram of particulate matter distribution across provinces. Both colored by province.

medRxiv preprint doi: https://doi.org/10.64898/2025.12.10.25341877; this version posted December 11, 2025. The copyright holder for this preprint (which was not certified by peer review) is the author/funder, who has granted medRxiv a license to display the preprint in perpetuity.

It is made available under a CC-BY 4.0 International license .

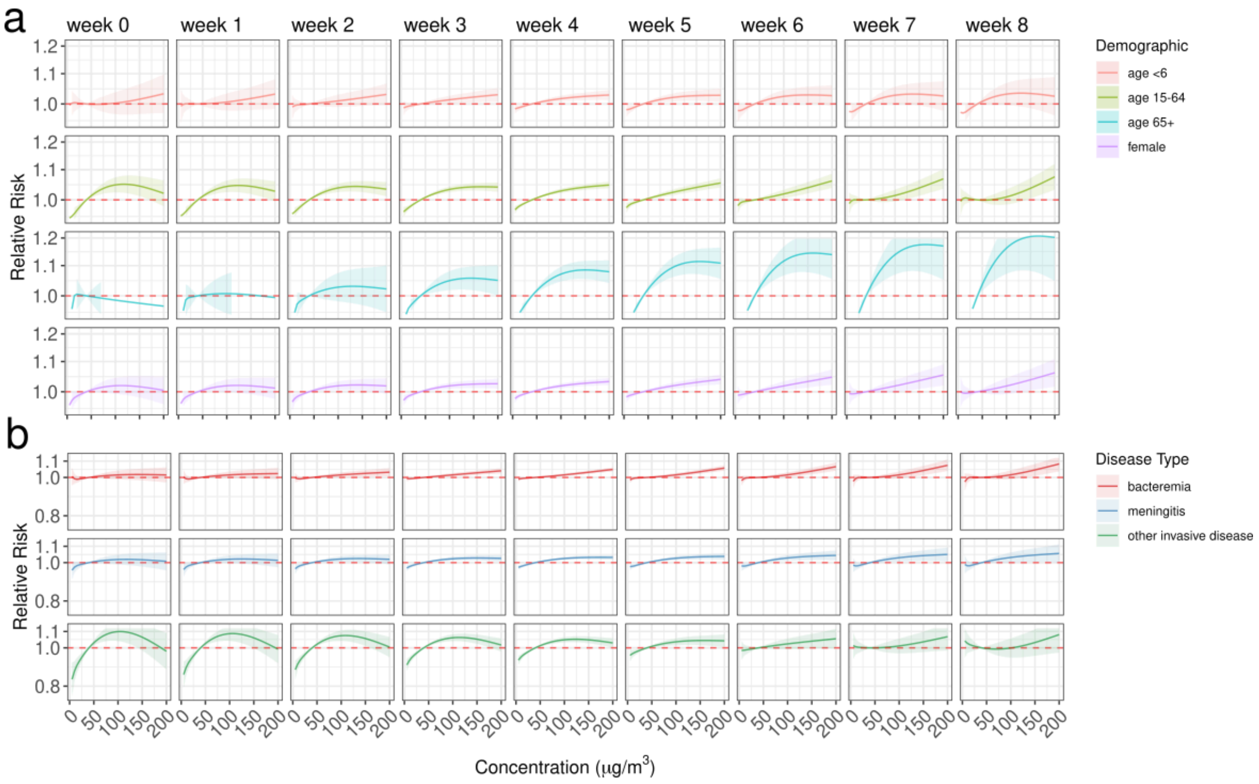

Figure S8. Exposure-response curves across different weekly exposure to $PM_{2.5}$ (concentration μg/m^3) including different stratifications of invasive disease including (a) demographic factors such as age <6 (red), age 15-64 (green), age >64 (blue), and females alone (purple) and for (b) different disease types including bacteremia (red), meningitis (blue), and other invasive disease types (dark green) all across 8 weeks of lags at the weekly district level.

medRxiv preprint doi: https://doi.org/10.64898/2025.12.10.25341877; this version posted December 11, 2025. The copyright holder for this preprint (which was not certified by peer review) is the author/funder, who has granted medRxiv a license to display the preprint in perpetuity.

It is made available under a CC-BY 4.0 International license .

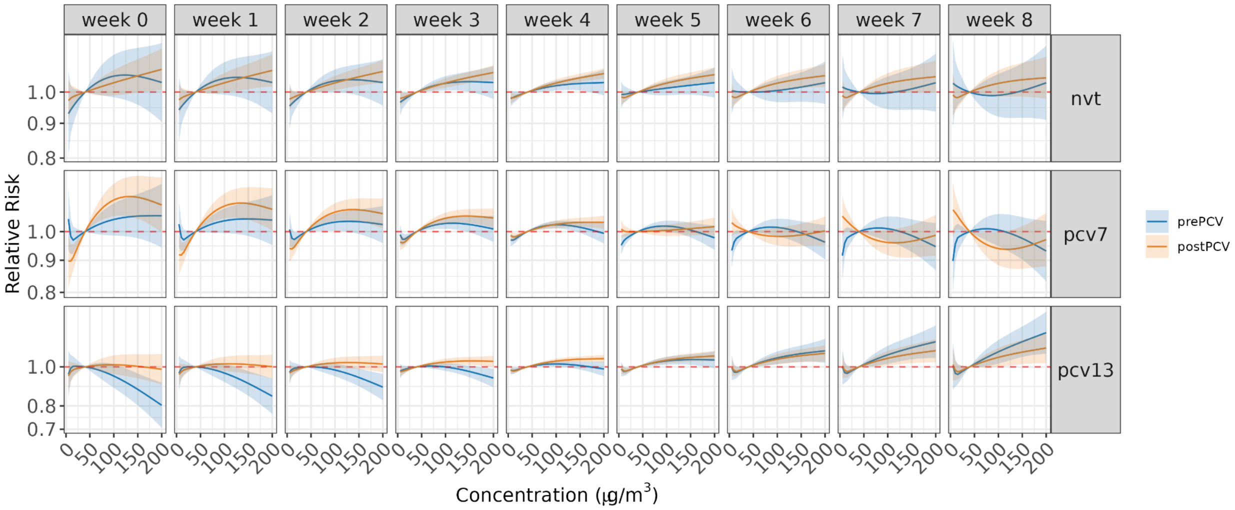

Figure S9. Exposure-response curves across different weekly exposure to $PM_{2.5}$ (concentration $\mu g/m^3$) including different stratifications of invasive disease including that which is caused by NVTs (top), PCV7 (middle), or PCV13 (bottom). These are further stratified by the pre-PCV period (2005-2009) (blue) and the post-PCV period (2009-2019) (orange) in a model run at the weekly-province level.

medRxiv preprint doi: https://doi.org/10.64898/2025.12.10.25341877; this version posted December 11, 2025. The copyright holder for this preprint (which was not certified by peer review) is the author/funder, who has granted medRxiv a license to display the preprint in perpetuity.

It is made available under a CC-BY 4.0 International license .

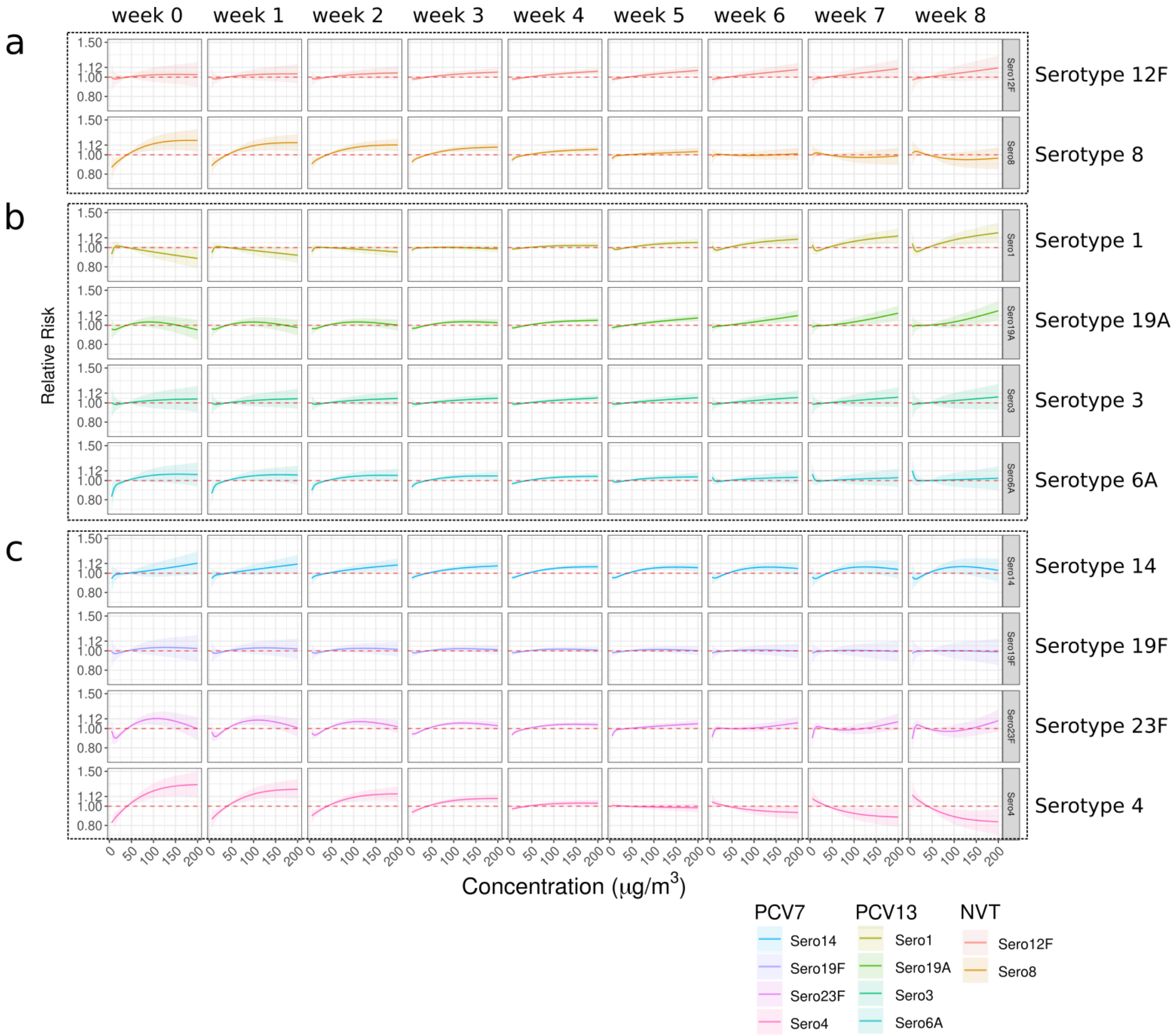

Figure S10. Exposure-response curves across different weekly exposure to $PM_{2.5}$ (concentration μg/m^3) including serotype stratifications of invasive disease including (a) NVTs serotype 12F and serotype 8, (b) PCV13 serotype 1, serotype 19A, serotype 3, serotype 6A, and (c) PCV7 serotype 14, serotype 19F, serotypes 23F, and serotype 4.

medRxiv preprint doi: https://doi.org/10.64898/2025.12.10.25341877; this version posted December 11, 2025. The copyright holder for this preprint (which was not certified by peer review) is the author/funder, who has granted medRxiv a license to display the preprint in perpetuity.

It is made available under a CC-BY 4.0 International license .

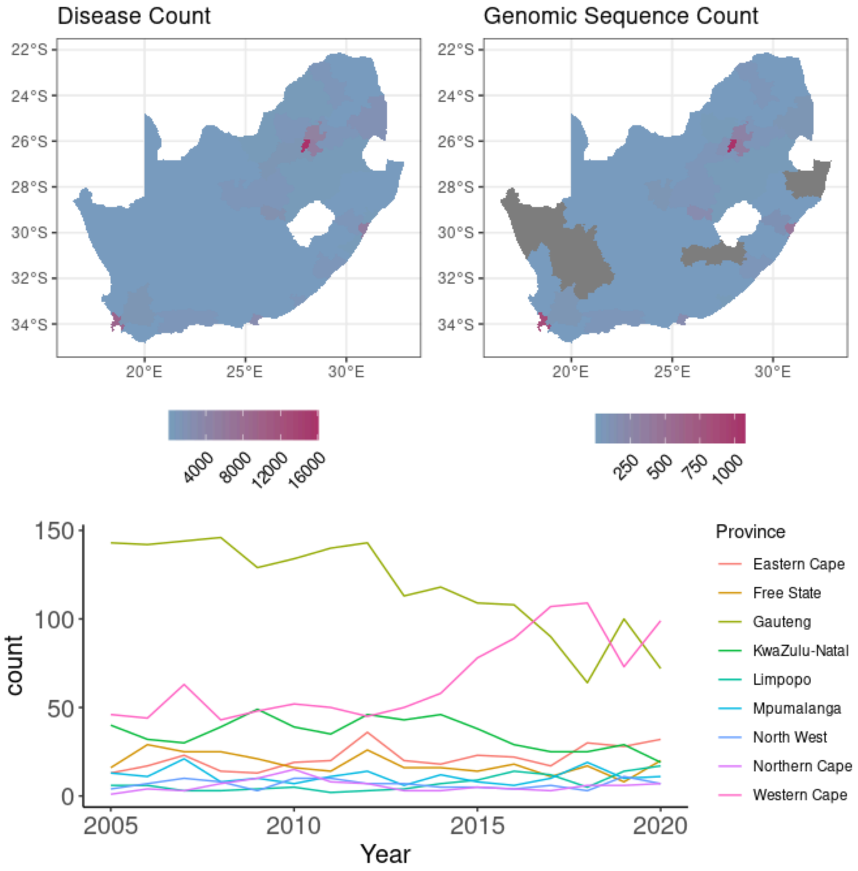

Figure S11. Summary of GPSC sequences as compared to case count and by province (a) Number of disease cases from each district and (b) the number of genomic sequences from each district. There were no sequences from 4 districts including: Namakwa, Joe Gqabi, uMkhanyakude, Zululand. (c) The number of pneumococcal samples sequenced from each province over time.

medRxiv preprint doi: https://doi.org/10.64898/2025.12.10.25341877; this version posted December 11, 2025. The copyright holder for this preprint (which was not certified by peer review) is the author/funder, who has granted medRxiv a license to display the preprint in perpetuity.

It is made available under a CC-BY 4.0 International license .

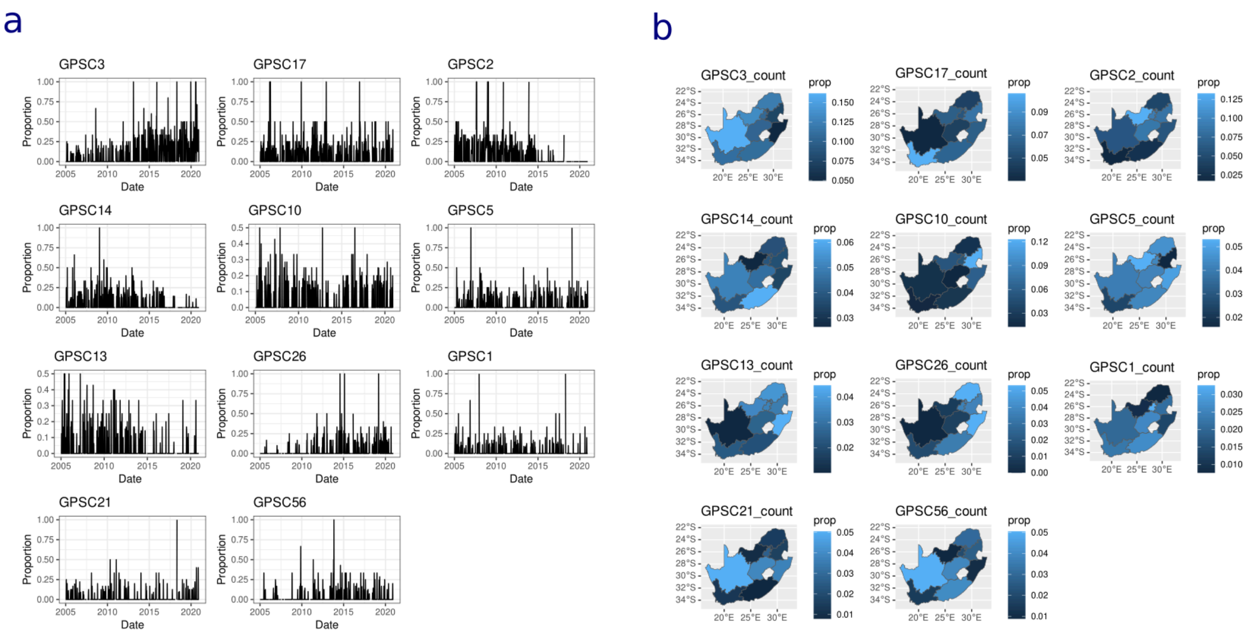

Figure S12. Proportion of each GPSC group annually and spatially per province in South Africa (N=9) across 15 years. The denominator is the number sequenced per week per province.

medRxiv preprint doi: https://doi.org/10.64898/2025.12.10.25341877; this version posted December 11, 2025. The copyright holder for this preprint (which was not certified by peer review) is the author/funder, who has granted medRxiv a license to display the preprint in perpetuity.

It is made available under a CC-BY 4.0 International license .

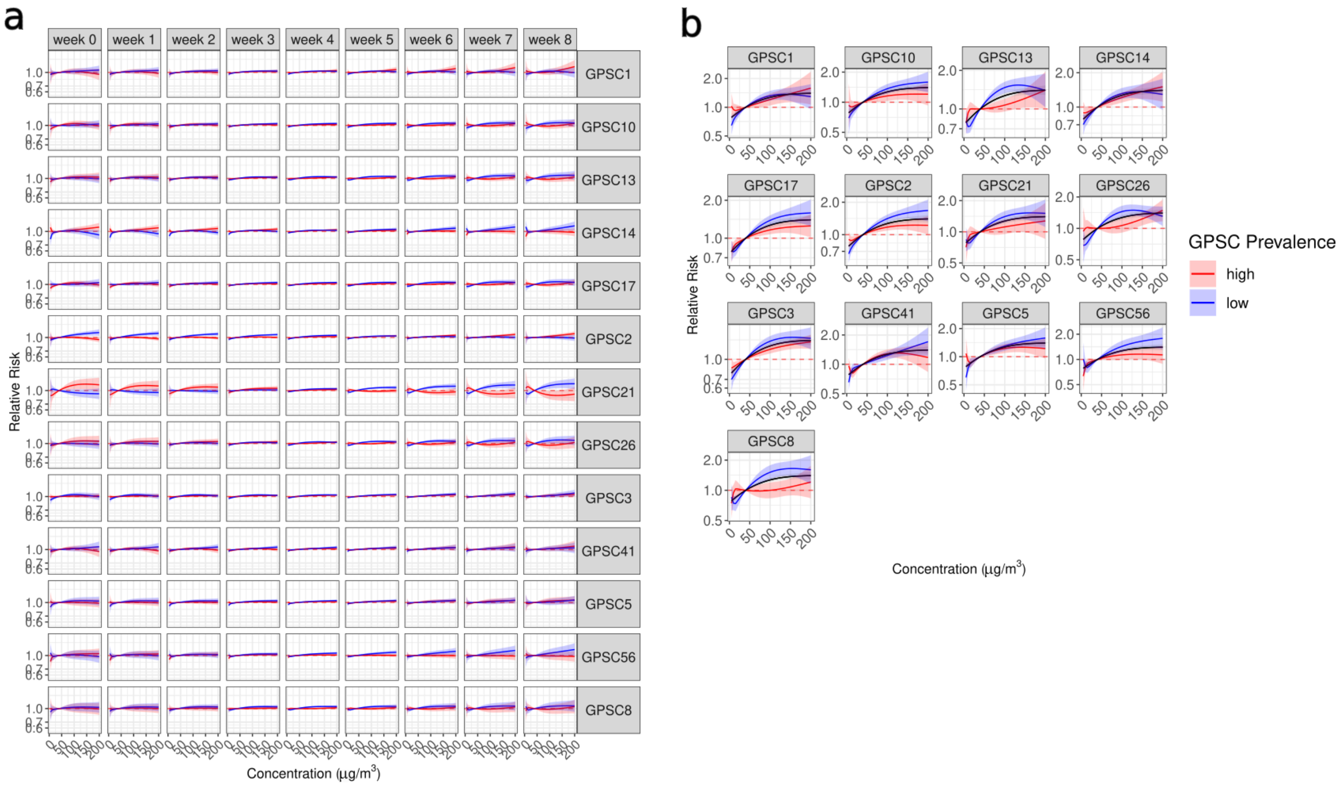

Figure S13. Exposure-response curves for $PM_{2.5}$ and pneumococcal disease interacted with the proportion of each GPSC per week. (a) 8-week lag time exposure-response curves and (b) includes the cumulative effect. The estimates are at high (red) and low (blue) GPSC prevalence. In b the black line indicates the model with no interaction.

medRxiv preprint doi: https://doi.org/10.64898/2025.12.10.25341877; this version posted December 11, 2025. The copyright holder for this preprint (which was not certified by peer review) is the author/funder, who has granted medRxiv a license to display the preprint in perpetuity.

It is made available under a CC-BY 4.0 International license .

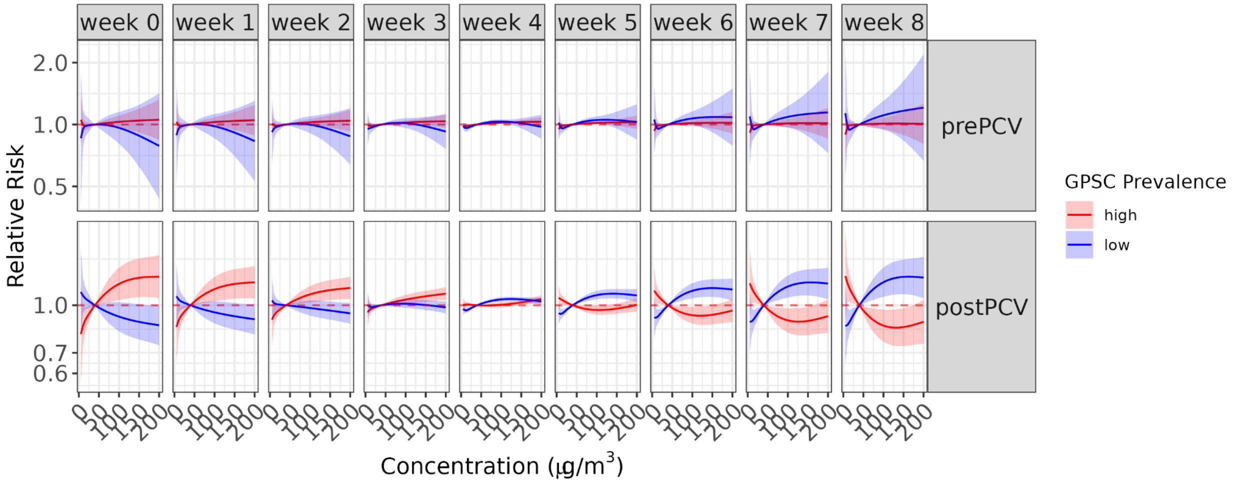

Figure S14. GPSC21-exposure-response curves for the relative risk of IPD given the concentration of $PM_{2.5}$ and interacted with the weekly proportion of GPSC21 in province level weekly models across an 8-week lag for (top) pre-PCV period 2005-2008 and (bottom) post-PCV period 2009-2019.

medRxiv preprint doi: https://doi.org/10.64898/2025.12.10.25341877; this version posted December 11, 2025. The copyright holder for this preprint (which was not certified by peer review) is the author/funder, who has granted medRxiv a license to display the preprint in perpetuity.

It is made available under a CC-BY 4.0 International license .

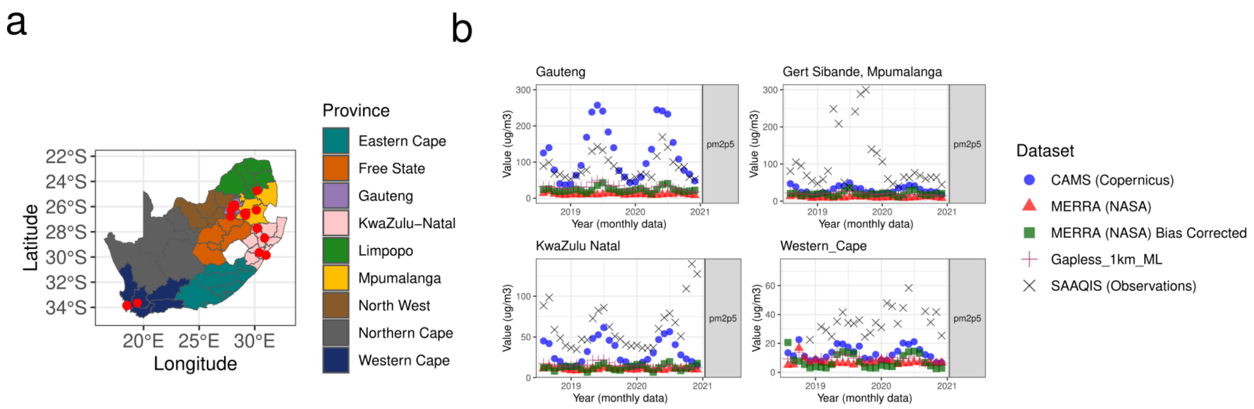

Figure S15. Reanalysis data compared to observations in South Africa. (a) Map of air quality monitoring stations included from SAAQIS. Colored by province with lines distinguishing districts. Locations of observation stations are indicated in red dots. (b) The concentration of weekly $PM_{2.5}$ in μg/m^3 from Gauteng across reanalysis products from Copernicus (CAMS), MERRA-2 from NASA, MERRA-2 with a bias adjustment and a machine learning gapless product at 1km grid squares.

medRxiv preprint doi: https://doi.org/10.64898/2025.12.10.25341877; this version posted December 11, 2025. The copyright holder for this preprint (which was not certified by peer review) is the author/funder, who has granted medRxiv a license to display the preprint in perpetuity.

It is made available under a CC-BY 4.0 International license .

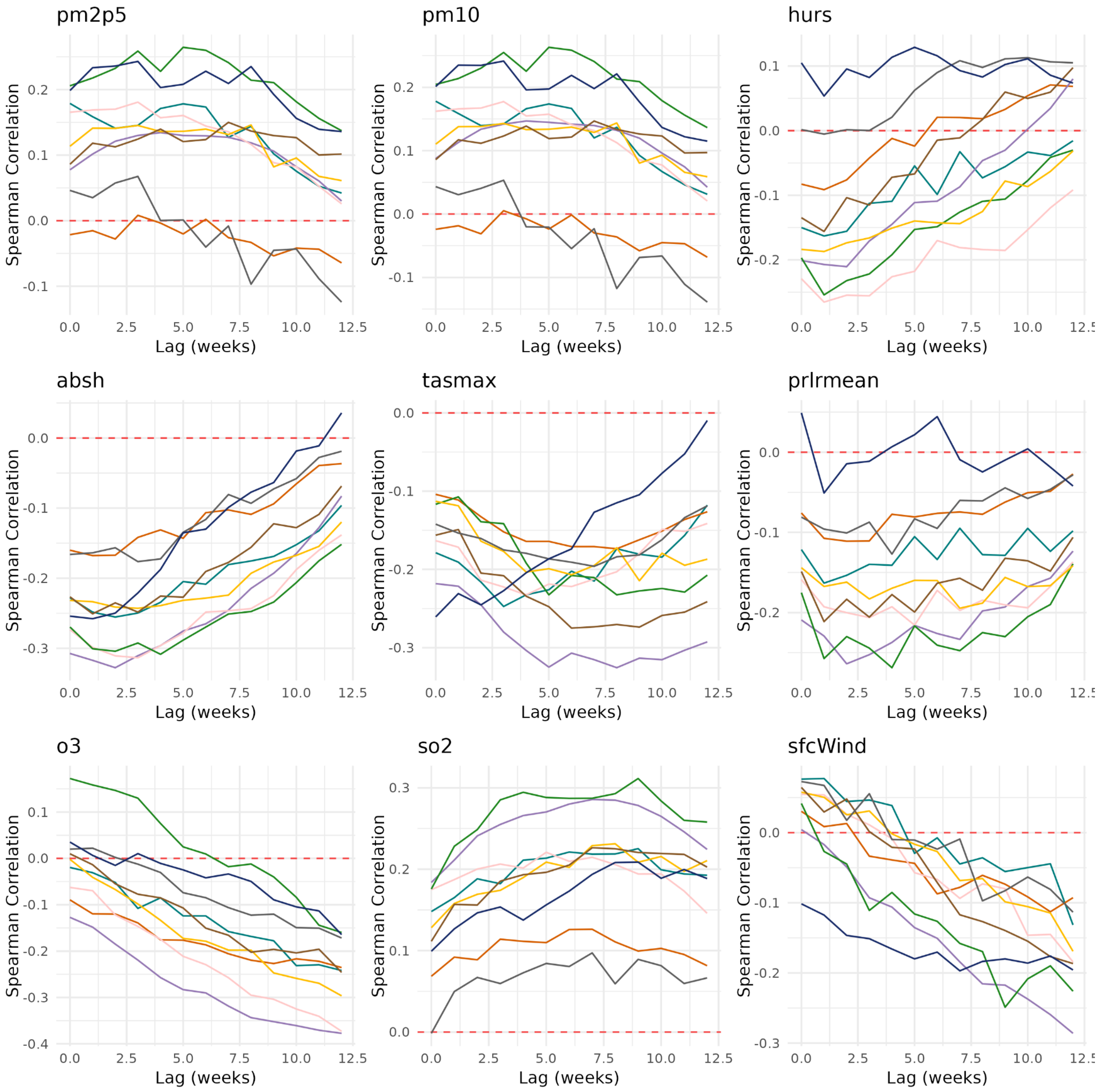

Figure S16. Spearman correlation between each lagged variable across lag weeks with disease case counts for all environmental variables colored by province.

medRxiv preprint doi: https://doi.org/10.64898/2025.12.10.25341877; this version posted December 11, 2025. The copyright holder for this preprint (which was not certified by peer review) is the author/funder, who has granted medRxiv a license to display the preprint in perpetuity.

It is made available under a CC-BY 4.0 International license .

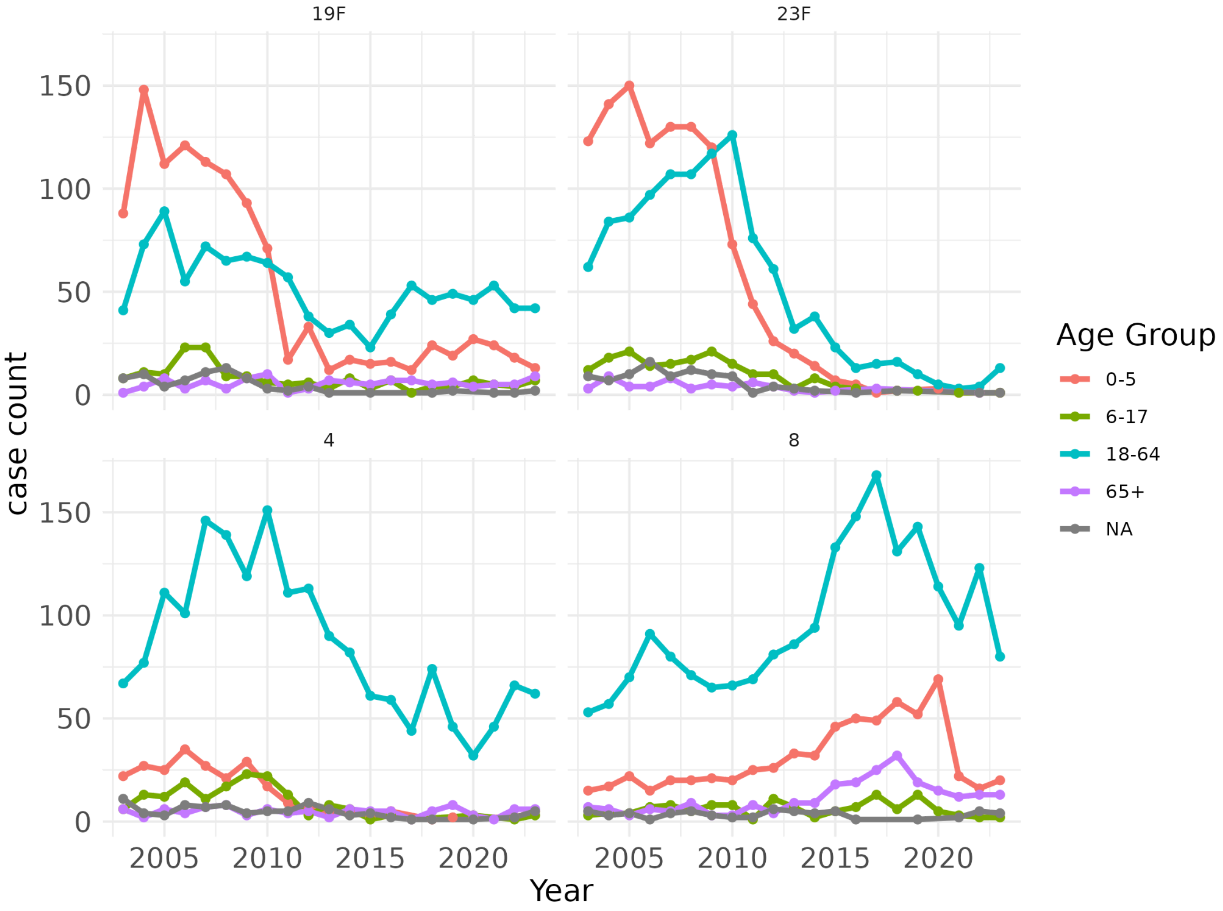

Figure S17. Proportion of IPD cases per age group and year by serotype. Number of IPD cases (y-axis) per year (x-axis) stratified by 0-5 (pink), 6-17 (green), 18-64 blue), and 65 and over (purple). Missing age data (grey). Stratified by PCV13 serotype 19F (top left), PCV13 serotype 23F (top right), PCV13 serotype 4 (bottom left), and NVT serotype 8 (bottom right).
